# Phages and HIV-1: From Display to Interplay

**DOI:** 10.3390/ijms13044727

**Published:** 2012-04-13

**Authors:** Sylvie Delhalle, Jean-Claude Schmit, Andy Chevigné

**Affiliations:** 1 Laboratory of Retrovirology, CRP-Sante, 84, Val Fleuri, L-1526 Luxembourg, Luxembourg; E-Mails: jc.schmit@crp-sante.lu (J.-C.S.); andy.chevigne@crp-sante.lu (A.C.); 2 Service National des Maladies Infectieuses, Centre Hospitalier Luxembourg, 4, rue E. Barblé, L-1210 Luxembourg, Luxembourg

**Keywords:** phage display, HIV-1, epitope mapping, mimotopes, HIV-1 inhibitor, HIV-1 vaccine, gp120, gp41, CXCR4, CCR5

## Abstract

The complex hide-and-seek game between HIV-1 and the host immune system has impaired the development of an efficient vaccine. In addition, the high variability of the virus impedes the long-term control of viral replication by small antiviral drugs. For more than 20 years, phage display technology has been intensively used in the field of HIV-1 to explore the epitope landscape recognized by monoclonal and polyclonal HIV-1-specific antibodies, thereby providing precious data about immunodominant and neutralizing epitopes. In parallel, biopanning experiments with various combinatorial or antibody fragment libraries were conducted on viral targets as well as host receptors to identify HIV-1 inhibitors. Besides these applications, phage display technology has been applied to characterize the enzymatic specificity of the HIV-1 protease. Phage particles also represent valuable alternative carriers displaying various HIV-1 antigens to the immune system and eliciting antiviral responses. This review presents and summarizes the different studies conducted with regard to the nature of phage libraries, target display mode and biopanning procedures.

## 1. Introduction

In 1983, the human immunodeficiency virus (HIV-1) was identified as the causative agent of the Acquired ImmunoDeficiency Syndrome (AIDS) [1,2]. In 30 years of pandemic, HIV-1 has infected more than 60 million individuals and killed 25 million. Thirty-three million individuals are currently living with HIV-1 making this disease a major worldwide public health problem (UNAIDS 2010). Natural sterilizing immune response against HIV-1 has never been described and despite decades of intensive research, a vaccine against HIV-1 is still lacking, mainly due to the high ability of the virus to escape from the immune response.

In the absence of a vaccine, combinations of small antiviral molecules are intensively used to control HIV-1 infection. The majority of these drugs are reverse transcriptase and protease inhibitors [3]. More recently, new molecules targeting the fusion step, CCR5 or integrase were licensed for clinical use [4–6]. Despite the increased life expectancy observed with the advent of these therapies, severe side effects, lack of adherence and emergence of drug-resistant virus strains still limit the long-term control of the infection [7].

HIV-1 is an enveloped virus whose genetic material consists of two identical RNA strands coding for the structural genes *gag*, *pol* and *env* as well as the accessory genes *tat*, *rev*, *nef*, *vif*, *vpr* and *vpu*. The *gag* gene codes for structural proteins p17 and p24, while *pol* codes for viral enzymes (reverse transcriptase, integrase and protease) and *env* for the gp160 envelope protein precursor that is subsequently cleaved into gp120 and gp41. Gp120 and gp41 proteins assemble at the surface of HIV-1 into trimeric spikes composed of three monomers of membrane-embedded gp41 complexed to free gp120. These two proteins are involved in virus entry and represent the principal targets for the humoral response.

**Figure 1:**
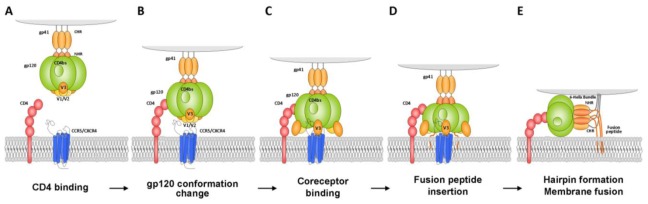
Model for HIV-1 entry. (**A** and **B**) Binding of Cluster of Differentiation (CD)4 to glycoprotein (gp)120 exposes a coreceptor binding site in gp120; (**C** and **D**) Coreceptor binding causes the exposure of the gp41 fusion peptide and its insertion into the membrane of the target cell in a triple-stranded coiled-coil; (**E**) Formation of a helical hairpin structure in which gp41 folds back on itself is coincident with membrane fusion.

Upon CD4 receptor binding, glycoprotein gp120 undergoes conformational changes exposing the V3 loop, a region that further interacts with the chemokine receptors CCR5 or CXCR4 thereby promoting viral entry [8] (Figure 1). Coreceptor binding leads to the insertion of the gp41 fusion peptide into the cell membrane, the creation of a hairpin loop intermediate and finally the fusion of both viral and cell membranes. The viral capsid then enters the cell and the genetic material is released in the cytoplasm. Most viral strains use only one coreceptor to enter host cells and are classified accordingly as CCR5- (R5 strains) or CXCR4-tropic (X4 strains), although viruses with broadened coreceptor usage (dual-tropic) have also been described. R5 viruses infect macrophages and CCR5-expressing T lymphocytes, and are mainly associated with transmission. In contrast, X4 viruses infect CXCR4-expressing T-cells and T-cell lines, and often appear at the later stages of infection.

The envelope glycoprotein gp120 is composed of variable and more constant regions. Several studies demonstrated that the elicitation or binding of effective neutralizing antibodies are impaired by the gp120 glycan shield or steric hindrance of its constant regions [9]. Moreover, variable immunodominant domains were shown to be recognized by non-neutralizing antibodies. Nonetheless, it is estimated that 10% to 30% of HIV-1-positive subjects develop neutralizing antibodies (NtAbs) appearing at least 1 year after infection. Only 1% of infected patients develop a broad neutralizing response against heterologous virus strains [10]. Among HIV-1-infected patients, such antibodies arise only rarely and tardily, thus inefficiently controlling viral replication. However, the recent identification of broadly neutralizing antibodies (BNtAbs) and mapping of their epitopes fueled interest in the humoral immune response against HIV-1 (reviewed by Overbaugh [11]).

To better understand the reasons underlying the persistance of viral infection despite the strong and sustained immune response on the one hand, and to identify new protective immunogens, numerous studies were conducted to map the epitope landscape of both HIV-1-neutralizing and non-neutralizing antibodies isolated from infected patients. In parallel, the development of new molecules or antibody fragments capable of blocking either viral proteins or host receptors has been widely investigated.

To serve this purpose, the phage display technology has been extensively exploited in the field of HIV-1 as it represents one of the most powerful technologies for epitope mapping as well as for the identification of ligand binding to many types of targets.

Bacteriophages (phages) are bacteria-infecting viruses whose DNA or RNA genome is packed in a capsid composed exclusively of surface proteins. The principle of phage display relies on cloning of exogenous DNA in fusion with the phage genetic material allowing the display of foreign peptides in an immunologically and biologically competent form at the surface of phage capsid proteins [12]. The significance of phage display was first demonstrated for filamentous phages such as M13, fd or related phagemids and later extended to lytic bacteriophages λ, T4 and T7 (reviewed by Beghetto [13]). The phage biopanning process consists of iterative cycles of binding, washing and elution steps leading to the progressive selection of phages displaying peptides/proteins binding to the target of interest [14]. The target is usually immobilized on a solid support which can be plastic, beads or even cells.

A significant advantage of this technology is that phages may be used to display a collection of sequences (phage library), reaching up to billions of distinct sequences. Phage libraries can be constructed to express combinatorial peptides or proteins/immunoglobulin fragments/variants that may be further screened for many different purposes including drug discovery, epitope mapping, diagnosis as well as identification of therapeutic antibodies. The phage display technology is versatile as it can be applied to different domains of research; it allows the easy handling and high-throughput screening of billions of sequences. It is affordable and enables the identification of linear as well as conformational epitopes when applied to an antibody target.

**Figure 2:**
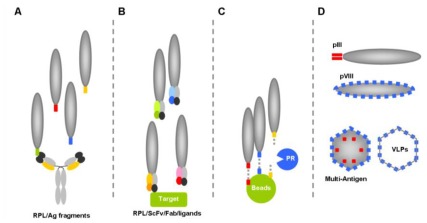
Phage display applications in the field of HIV-1. (**A**) Epitope mapping (section 1): Randomized Peptide Library (RPL) or antigen-fragment libraries are used to map the residues forming the epitope(s) recognized by either monoclonal or polyclonal antibodies immobilized on a solid support; (**B**) Inhibitor discovery (section 2): RPL, antibody-fragments or ligand variants libraries are used to select for viral or host-cell protein inhibitors; (**C**) Phage substrate (section 3): phage particles immobilized on a solid support are specifically eluted by the proteolytic activity of the HIV-1 protease; (**D**) Phage as carrier (section 4): phage particles or derived Virus-Like Particles (VLPs) are used to display immunogenic peptides or whole antigens to the immune system.

Numerous studies have described the use of the phage display technology in the field of HIV-1 were reported. They can be classified in four main applications (Figure 2):

- Epitope mapping, which relies on the screening of random peptide libraries on immobilized monoclonal or polyclonal antibodies to determine the linear and/or conformational epitopes recognized by these antibodies (linear epitope: sequence of continuous amino acids recognized by the paratope of a given antibody; conformational/discontinuous epitope: group of amino acids scattered along a protein sequence which come together in the folded protein and are recognized by the paratope of a given antibody). Such screening usually results in the identification of sequences mimicking the natural epitope (mimotopes) and provides precise information about the location of residues forming the natural epitope. These mimotopes may in turn be used as valuable immunogens to elicit antibodies targeting the original epitope, an approach referred to as “reverse vaccinology”.
- Inhibitor discovery, based on screening of phage libraries displaying random peptides or antibody fragments against viral or host proteins critical for viral replication.
- “Phage Substrate” approach in which potential substrate sequences are displayed at the phage surface to enzymes such as proteases. This approach not only allows for proteolysis specificity profiling, but provides information and data to develop specific inhibitors.
- Carrier phage, in which the phage functions as a “carrier”, “vehicle” or “virus-like particle” to display exogenous peptides such as mimotopes or even full size antigens to the immune system, to elicit specific humoral and/or cytotoxic T-cells responses.

This review is intended as an overview of the different studies conducted using phages in the field of HIV-1, laying special emphasis on the nature of the phage libraries used, the target display mode, the biopanning procedure as well as the results obtained. These studies are classified according to the 4 applications described above and the main results are presented in tables.

## 2. Exploration of HIV-1 Epitope Landscape

Monoclonal antibodies (MAbs) or polyclonal antibodies (PAbs) epitopes can be identified through screening either combinatorial or antigen-fragment libraries displayed at the surface of phages. Antibodies may be derived from infected/immunized animals or from HIV seropositive patients with peculiar immunological profiles, such as the Long Term Non Progressors (LTNP) [15], leading to the identification and characterization of HIV mimotopes.

### 2.1. Antibodies Directed against Viral Proteins

#### 2.1.1. Monoclonal Antibodies Directed against Viral Epitopes

##### 2.1.1.1. Gp120 V3 Loop

**Figure 3:**
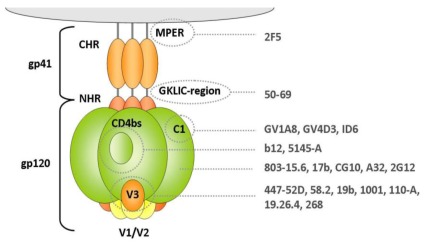
Epitope landscape of HIV-1 Env proteins. Monoclonal antibodies were mapped to different sites on the surface of the gp120 and gp41 proteins.

The seminal paper characterizing the epitope recognized by a MAb directed against HIV-1 using the phage display technology was published in 1993. Keller *et al.* screened a 15-mer Random Peptide Library (RPL) against the BNtAb 447-52D which targets the V3 loop of gp120 (KRKRIHIGPGRAFY) [16] (Figure 3) and identified 70 clones presenting a GPxR consensus sequence [17] (Table 1). These mimotopes were further used in rabbit immunization experiments and elicited neutralizing responses. Boots *et al.* later investigated the linear epitope recognized by the MAb 447-52D by combining gp120 competition and panning of V3-region biased/constrained libraries. Such a set-up favors the selection of mimotopes in which residues surrounding the GPGR crown motif are similar to those present in the gp120 used for competition, suggesting that the use of strain-specific competitors with a MAb of broad specificity can select for strain-specific mimotopes [18].

In the same year, Jellis *et al.* applied the phage display technology to the MAb 58.2, which recognizes the V3 loop of the HIV-1 MN strain [36,37]. In their study, a 20-mer RPL was constructed and panned against MAb 58.2 according to two different protocols, either streptavidin capture of phages mixed with biotin-labeled MAb 58.2 (SA-Bio) or panning against MAb 58.2 immobilized on microwells (micropan) [19]. Phages selected with the SA-Bio protocol shared a consensus sequence (Y/L)(V/L/I)GPGRxF homologous to the V3 loop. The micropan protocol allowed for the identification of sequences sharing the same motif, of which two were also identified in the SA-Bio panning. Biopanning results were further validated in peptide array hybridization assays. Hybridization of MAb 58.2 to 14-mer peptides containing all possible point substitutions within the V3 loop sequence demonstrated that both phage display and peptide array experiments identified the same critical amino acids, thereby confirming the quality of the 20-mer RPL and the validity of the screenings performed.

Epitope mapping was also performed on a monoclonal antibody (MAb 19b) isolated from an asymptomatic HIV-1-infected patient and recognizing the xxIx_3_PGRAFYTT motif within the V3 loop sequence (KRIHIGPGRAFYTT) [38]. Binding of MAb 19b to viral isolates presenting mutations in this sequence revealed that not all residues within this recognition motif were crucial for reactivity [20]. Biopanning with a 15-mer RPL resulted in the selection of sequences compatible with the minimal binding site (-I----G--FY-T) inferred from gp120 sequence alignment from clades A to F which bound MAb 19b. Taken together, data from binding assays as well as phage biopanning experiments demonstrated that the MAb 19b epitope spans both sides of the V3 loop. Substitutions within the residues located at the crown of the loop are however tolerated, provided that the formation of a β-turn induced by the GPGR crown motif is allowed. However, one exception was reported by Boots *et al*. who reported that the Phe to Trp substitution may be tolerated in the absence of a β-turn [18].

In parallel, Grihalde *et al.* constructed and panned a 30-mer RPL against MAb 1001, which recognizes a constrained linear epitope on the V3 loop [21]. Several clones were obtained and presented the common motif (R/K/H)xGR mimicking the crown of the V3 loop sequence, thereby confirming the epitope sequence of MAb 1001. To assess the reactivity of peptides deprived of the phage scaffold, the mimotope with the highest affinity for the MAb 1001 was expressed in fusion with the *E. coli* alkaline phosphatase. Binding of the phage and fusion protein to the MAb 1001 was assessed by ELISA, Western Blot and SPR assays and highlighted that binding was independent from the scaffold, although interactions were weaker when the peptide was displayed in the fusion protein format than in the phage scaffold.

In another study, Laisney *et al.* investigated the minimal epitopes recognized by two MAbs interacting with the V3 loop, 110-A and 19.26.4, whose specificity is strictly restricted to the X4-tropic LAI isolate [22]. The screening of a 6-mer RPL on the MAb 110-A allows the selection of numerous sequences with a consensus motif. Binding assays with synthetic peptides further showed that both MAbs reacted with residues 316–320 of the LAI gp120. In this narrow region, the minimal epitope deduced for the MAb 110-A was Hyx**R**GP, whereas the MAb 19.26.4 recognized the x**Q**(**R**/K)GP motif (Hy: non-aromatic AA, underlined: AA tolerating substitutions). Interestingly, the essential **QR** residues located at positions 317–318 correspond to a QR insertion located upstream of the V3 loop GPGR crown motif that is characteristic of the LAI isolate and may thus explain the restricted specificity of the two MAbs. The same authors screened a 6-mer RPL on the MAb 268, specific to the V3 loop of the MN isolate, and identified two groups of sequences [23]. A representative sequence from the first group (268.1, HLGPGR), corresponded to the crown of the V3 loop, a linear epitope, while two sequences of the second group (268.2, KAIHRI and 268.3, KSLHRH), showed no homology to linear HIV-1 epitopes. Both peptides 268.1 and 268.2 nevertheless inhibited the interaction of MAb 268 with gp120, and were even able to compete with each other for binding to the antibody, indicating that peptide 268.2 was also a mimotope of peptide 268.1. When conjugated to KLH and injected separately into rabbits, both peptides 268-1 and 268-2 were able to elicit gp120-reacting antibodies that partially competed with the homologous peptide, confirming that 268.1 and 268.2 peptides are both antigenic and immunogenic mimics of the gp120 MN V3 loop.

##### 2.1.1.2. Gp41 Membrane Proximal External Region (MPER)

The isolation of the BNtAb 2F5, which interacts with an epitope (ELDKWA) located on the gp41 MPER was reported in 1993 [39]. Conley *et al.* further characterized this epitope by biopanning a 15-mer RPL on immobilized 2F5 Ab. Different sequences were obtained and classified in four groups, whose consensus motifs, **DKW**, L**D**x**W**, E**D**(**K**/R)**W** and EL**DKW**, revealed information on the residues involved in 2F5 Ab recognition [24].

Immunization attempts with ELDKWA peptides failed to elicit 2F5-like NtAbs, suggesting that the epitope necessitates additional residues in order to be immunogenic. Therefore, Menendez *et al.* screened a panel of 17 libraries of linear and constrained peptides against the MAb 2F5 and deduced that residues flanking the **DKW** core at the *C*-terminal side region were important for high-affinity binding to the MAb [25]. They subsequently constructed and screened two phage sublibraries displaying 12 random residues either upstream or downstream of the DKW core (x_12_-AADKW and AADKW-x_12_) and isolated three peptides displaying high affinity for 2F5 from the AADKW-x_12_ library. Ala substitution and deletion studies revealed that each clone bound 2F5 according to a different mechanism. This data led the authors to postulate that the 2F5 paratope was composed of two binding domains either recognizing the DKW core with strong specificity or multispecifically binding to the residues located at its *C*-terminus.

Based on this study, additional investigations were recently conducted on the BNtAb 2F5 epitope to assess the importance of structural constraints for MAb 2F5 recognition [26]. A linear 12-mer RPL and a constrained 7-mer RPL were screened against this antibody and all the sequences selected from the 12-mer RPL contained the D(K/R)W core motif, with flanking residues L, A and S present at different frequencies. Analysis of the sequence representation compared to their estimated probability of occurrence indicated a trend towards enrichment for sequences such as DKWA or LDKWA throughout panning of the 12-mer library, while all sequences selected from the constrained library contained DKWA or LDKWA. These results demonstrated that the strong epitope specificity postulated by Menendez [25] is only displayed when the epitope sequence is presented in a certain structural context provided in the constrained 7-mer peptides. Immunization studies performed with both linear and constrained forms of the peptide in mice and rabbits resulted in the inhibition of cell fusion only with sera of rabbits immunized with the linear peptide.

##### 2.1.1.3. Gp120 C1 Domain

RPL screening may also contribute to the elucidation of the antigen structure. To that purpose, Stern *et al.* used a 20-mer RPL to analyze two different mouse MAbs (GV1A8 and GV4D3) recognizing non-overlapping sequences between residues 1 and 142 of gp120 [27,40]. Biopanning performed on GV1A8 allowed for identification of mimotopes sharing a (L/I)W motif identical to residues 111–112 of the gp120 C1 domain and highlighted a HxxIxxLW motif compatible with two turns of an α-helix. Computer modeling confirmed that such a structure placed the residues recognized by GV1A8 contiguously on one face of the helix while other secondary structures did not. Similarly, biopanning on MAb GV4D3 yielded sequences with a trend towards an Nx_3_WxxD motif. The epitope maps to the FNMWKND sequence satisfying the helical motif FxxWxxD. In this study, the use of phage display not only predicted the α-helix structure of the C1 domain of gp120, but also pinpointed the contact residues defining the surface of the helix.

Phage display was also applied to epitope mapping of Antibody-Dependent Cellular Cytotoxicity (ADCC)-inducing MAbs since HIV-1 infected cells may be targets for Fc receptor-bearing effector cells interacting with HIV-1-specific Abs. Screening of 7-mer, 7-mer-c and 12-mer RPLs against the ADCC-inducing MAb ID6 resulted in the identification of phages with TxxFxxWxxD (12-mer RPL) and FxDWxF (7-mer and 7-mer-c RPLs) motifs homologous to the C1 domain of gp120 [28].

Competition assays showed that binding of MAb ID6 to gp120 or gp160 was abrogated in the presence of 12-mer mimotopes. In contrast, heptapeptide mimics only slightly impaired this binding, supporting the hypothesis that the MAb-ID6 epitope probably encompasses residues additional to the FxDWxF motif. As this epitope is highly conserved among circulating HIV-1 subtypes, it might be useful to induce MAb ID6-like antibodies.

##### 2.1.1.4. Gp120 CD4-Binding Site

The BNtAb IgG1 b12 was the first neutralizing MAb selected from a phage-displayed Fab (antibody fragment composed of one constant and one variable domain of the heavy (CH1 and VH) and the light (CL and VL) chains linked together) library derived from an HIV-1-infected donor (See section 3.1.1.1.1.) [41]. This antibody recognizes a conformational epitope overlapping the CD4-binding site of gp120 [42]. Attempts to precisely map the residues interacting with the IgG1 b12 MAb with 15-mer and 21-mer RPLs provided no consensus sequence [18]. As previous screening of 11 cysteine-enriched peptide libraries resulted in the identification of two sequences bearing an SDL motif flanked by one or two cysteine residues (REKRWIFSDLTHT**C**I and T**C**LWSDLRAQ**C**I) [30], Zwick *et al.* constructed two sublibraries (x_7_**SDL**x_3_**CI and** x**C**xx**SDL**x_3_**CI)** sharing the SDL motif and reflecting the cysteine content of the two clones [29]. A B2.1 peptide (HERSYMF**SDL**ENR**CI)** containing a unique cysteine bound b12 in Fab as well as IgG1 formats with a much higher affinity than the other clones. Moreover, the phage-borne B2.1 peptide was used to screen the Fab library from which b12 was identified. This “reverse panning” experiment showed that B2.1 was able to select only the Fab sequence corresponding to b12, confirming the specificity of the B2.1 mimotope towards the b12 Ab. B2.1 peptide was immunogenic in mice and rabbits but did not elicit significant anti-gp120 cross-reactive Abs titers.

Dorgham *et al.* attempted to map the b12 epitope with a RPL of two random 10-mers joined through an ALLRY spacer (x_10_ALLRYx_10_) [31]. Selection resulted in the identification of clones sharing a M/VArSD consensus motif (Ar standing for any aromatic residue) as previously observed [29]. A second- and a third-generation of semi-RPL containing fixed consensus motifs identified in the previous panning surrounded by randomized residues were constructed (x_3_(M/V)WSDx_3_ and xLXVWxDExx). Phagotopes (phage particle displaying a particular peptide sequence selected on a given target) obtained from the first, second and third generation libraries showed increasing binding affinity for b12, respectively. Phagotopes were able to compete with gp160 for b12 binding and triggered the production of Abs capable of recognizing at least five distinct, unrelated HIV-1 strains. In contrast, the corresponding peptides were not able to compete for b12 binding and did not elicit anti-gp160 MAbs. Such discrepancies between phagotopes and peptides might be explained by constraints imposed by the phage scaffold.

Detailed characterization of the BNtAb b12 was conducted with the Mapitope algorithm developed by Enshell-Seijffers *et al.* to facilitate the identification of discontinuous epitopes. This approach is based on the assumption that the collection of mimotopes recognized by a given antibody must in some manners reflect the antibody’s paratope [32]. A constrained 12-mer RPL was screened against b12 and selected sequences were compared to those obtained from previous panning experiments performed against b12 [18,29,30,43]. Although no similarity was observed with the mimotopes selected by Boots *et al.* [18], a consensus WSDL motif was observed in the newly identified mimotopes and the sequences isolated by Bonnycastle *et al.* [29,30]. Mapitope analysis conducted on these sequences as well as on the peptide sets isolated by Boots and Bonnycastle resulted for each of the three panels in the prediction of two clusters located at the periphery of the CD4 binding site.

At the same time, both a linear 9-mer RPL and a constrained 10-mer RPL were used in panning experiments against another gp120 CD4 binding site MAb (5145A) [33]. Screening of the 9-mer RPL resulted in selection of a single sequence (WKPVVIDFE), while screening of the 10-mer-c RPL on 5145A allowed for identification of a GPxEPxGxWxC consensus motif. Peptides were synthesized as peptide-pIII fusion proteins and their affinity for 5145A was assessed in phage/MAb and gp120/MAb binding inhibition assays. The two most affine peptides (AECGPAEPRGAWVC and AECGPYEPRGDWTCC) were used to immunize rabbits and elicited antibodies binding to recombinant monomeric gp120. Nevertheless, generated Abs seemed to target a different epitope since they were unable to compete with the 5145A CD4-binding site specific MAb.

##### 2.1.1.5. Other Domains

The extreme *C*-terminus of gp120 forms a pocket which may interact with gp41 and was suggested to undergo conformational changes weakening the interaction between gp120 and gp41 upon CD4 binding. In the absence of available crystallographic information, Ferrer *et al.* utilized the mouse MAb 803–15.6 to analyze an epitope overlapping with this pocket region [34]. Epitope mapping of MAb 803–15.6 achieved by cross-blocking experiments on gp120 suggested that the Ab recognized residues 502–516 while the screening of an heptapeptide RPL against MAb 803–15.6 preincubated with gp120 allowed for the recovery of phages presenting an AxxKxRH motif homologous to residues 502–508. Affinity studies confirmed that Ala was the *N*-terminal residue of the MAb 803–15.6 epitope and showed that affinity increased when *C*-terminal residues were added.

The Mapitope algorithm designed by Enshell-Seijffers *et al.* was initially developed to elucidate the CD4-induced epitope recognized by the MAb 17b [32]. Screening of a 12-mer-c RPL yielded sequences with no homology to gp120. Comparison of the mimotopes to the gp120 structure in complex with MAb 17b and sCD4 predicted candidate epitopes that were in agreement with the actual 17b contact residues. For further validation of the algorithm, RPL libraries were screened against the p24-specific MAb 13b5 and analysis of the selected sequences predicted four clusters, the largest of which corresponded to the genuine epitope. The algorithm was finally applied to the Mab CG10, an Ab with an unknown epitope competing with the Mab 17b for the binding to the CD4/gp120 complex. Mimotopes sequences were analyzed and produced seven clusters, one of them being in accordance with previous mutation analysis impeding Mab CG10 binding [44]. Noteworthingly, when reconstituted in a phage scaffold, the epitope was capable of binding Mab CG10.

After having successfully identified linear or nearly linear epitopes [17,20,24], Boots *et al.* extended the use of the phage display technology to the identification of epitopes recognized by MAbs binding to discontinuous sequences [18]. One of these Abs (MAb A32) binds to a CD4-induced discontinuous epitope involving residues within the C1, C2 and C4 regions of isolates from clades B, C, D, E and F [45,46]. Panning of a 15-mer RPL yielded several phages which only shared a Trp residue. In the same study, panning of a 15-mer RPL against MAb 50–69, which reacts with the ID GKLIC region of gp41, resulted in the identification of sequences sharing a common Trp within motifs **W**GCx(K/R)xLxC and FGx**W**FxMP. The selected consensus sequences were however not further characterized.

The BNtAb 2G12 presents the typical feature of recognizing a cluster of high-mannose oligosaccharides of gp120 [47–49]. In an attempt to identify peptidic immunogens capable of eliciting 2G12-like Abs, Menendez *et al.* screened a set of previously described RPLs [30] against 2G12 and identified one phagotope specifically binding to 2G12 (2G12.1) [35]. The crystal structure of MAb 2G12 complexed to the synthetic 2G12.1 peptide was compared to structures of 2G12-oligomannose epitopes and revealed that interactions with the Abs were different for the two ligands. These results showed that the peptide selected from RPL panning experiments is not a structural mimic of the 2G12 oligomannose epitope. The phagotope 2G12.1 was used in rabbit immunization experiments and elicited high titers of peptide-specific antibodies, but no cross-reactivity with gp120 was obtained, further supporting that peptide 2G12.1 is not an immunogenic mimic of the MAb 2G12 epitope.

#### 2.1.2. Polyclonal Antibodies Directed Against Viral Epitopes

The first attempt at identifying epitopes recognized by HIV-specific PAbs was performed in 1999 on plasma IgG from two LTNP patients (Table 2). Using linear and constrained 9-mer RPLs, Scala *et al.* identified mimotopes of the linear immunodominant (ID) GKLIC region of gp41 or the V1 and C2 domains of gp120 [50]. These mimotopes were immunogenic when injected to mice and elicited an NtAb response against HIV-1. Moreover, the same mimotopes reduced viraemia to undetectable levels in immunized monkeys as shown in a subsequent study [51]. The same year, a similar study conducted on one LTNP with an RPL library of cysteine-constrained 12-mers selected for peptides defining the gp41 ID epitope CSGKLIC. The levels of reactivity of these phagotopes were further assessed against a panel of HIV positive plasma to evaluate the plasticity and polyclonality of the immune response mounted by 30 infected individuals [52].

Later, Palacios-Rodriguez *et al.* evaluated the impact of factors such as Highly Active AntiRetroviral Treatment (HAART) or Ab titers on a selection of peptides mimicking the ID epitope CSGKLIC [53]. In their study, a mix of linear 12-mer as well as linear and constrained 7-mer RPL was screened against the individual plasma samples of four HIV-1 infected patients initiating HAART and presenting different titers of anti-GKLIC antibodies. A consensus motif CxxKxxC was obtained from the 12-mer linear RPL, and the percentage of occurrence of the motif in the selected sequences was proportional to the anti-GKLIC Ab titers of each sample, indicating that these Abs are involved in selection of the consensus motif. Mice immunization experiments with the two mimotopes resembling most to the gp41 ID parental epitope as well as with pools of phages eluted from the panning experiments showed that all phages elicited reactivity, and that immunization with the phage eluates induced the strongest recognition. These findings indicate that the immunogenic properties of mimotopes are different and additive, opening the possibility of immunizing animals with different mimotope combinations (See Section 4).

In 2007, Humbert *et al.* investigated the immune response of eight LTNP patients presenting BNtAbs. By using linear and constrained RPLs they identified epitopes recognized by plasma IgGs captured on tosylactivated beads [54]. Each panning round consisted of a positive selection performed on LTNP IgGs followed by a negative selection on the IgGs of healthy donors. Homologies of some selected sequences to immunodominant regions such as the gp120 V3 loop or the gp41 GKLIC region were observed, as reported in previous studies [50,52,53]. Further homologies to linear motifs located near the V3 loop (NNNT), downstream of the ID GKLIC region (AVPW motif) and overlapping with the 2F5 BNtAb epitope (PPWx_3_W motif) were also identified. Additionally, the authors applied the 3DEX software to compare the phage insert sequences to HIV-1 protein structure files from the RCSB Protein Data Bank (www.pdb.org) [59,60]. Phage pools corresponding to the linear V3 loop, GKLIC domain and WxxxW motif, as well as pools representing potential conformational epitopes, were selected for mice immunization assays, and elicited plasma-associated neutralizing activity against primary HIV-1 strains. The highest neutralizing ability was obtained with mice immunized with the V3 mimotopes, although immunization with potential conformational epitopes also provided a modest neutralizing response.

A similar approach was used by the same authors on a rhesus macaque infected with an SHIV chimera encoding the *env* of a clade C HIV-1 strain (SHIV1157ip) and presenting a broad neutralizing response against homologous SHIV-C as well as heterologous HIV-1 strains of different subtypes [61]. Biopanning yielded clones similar to gp120 (V2 and V3 loops or *C*-terminal domain) or to regions of gp41 (ID GKLIC region, other ID regions and MPER domain) [55]. Remaining clones showed no significant homology to linear HIV-1 regions and were analyzed with the 3DEX software, which allowed the identification of a discontinuous mimotope located near the V3 loop crown. The antibodies binding to this phagotope were affinity-purified and subsequent assays demonstrated that recognition was conformation-dependent.

An immunofocused immunization of mice primed with a DNA vector coding for the gp160 SHIV1157ip and boosted with pools of phage particles corresponding to the V3 loop, the gp120 *C*-terminus, the gp41 ID region, the GKLIC region and the MPER domain was set up. Almost all mice developed anti-env Abs and 59% of them presented a neutralizing activity.

In 2009, Dieltjens *et al.* applied the phage display technology to identify the epitopes potentially involved in the BNtAbs response of an HIV-1 CRF02AG-infected individual (ITM4) and to monitor the evolution of humoral response and viral escape through the course of infection [56]. Biopanning of a 12-mer RPL against plasma samples from ITM4 resulted in the identification of different peptide sequences. Half of these sequences were homologous to linear epitopes on gp41, *i.e.*, the 4E10 epitope region in the MPER domain (NWFNLTQTLMPR) or the lentivirus lytic peptide 2 (LLP2) (SLxxLRL) while the other peptides shared homologies with the C1 domain (KxWWxA) and the crown of the V3 loop (Kx_3_IGPHxxY) of gp120. Further analysis of the levels of reactivity of the phage groups against ITM4 six-year follow-up samples revealed different temporal patterns of recognition, confirming the dynamic nature of the immune response. Interestingly, the MPER region was the only epitope retaining immunogenic properties during this period.

In a more recent study, the same group investigated the antigenic landscape of an HIV-1 subtype A-infected individual with BNtAbs by screening an RPL library against a pool of sequential samples drawn from 1994 to 2005 [57]. The biopanning procedure yielded sequences predicted to represent autologous V2 sequence (Kx_3_Hx_3_Y), V3 loop (KxxHxGPx_3_F) and gp41 ID domain (CxGxLxCTxNxP). Again, follow-up sample recognition of the four phage groups showed different patterns. Antibody reactivity towards gp41 ID region fluctuated slightly in all plasma samples. Reactivity against the V3 loop-like phages decreased over time. In contrast, the V2 loop mimotopes were not recognized before 2001, but once emerged, reactivity persisted until 2005. Env sequence analysis of the follow-up samples showed that a Tyr to His mutation in the V2 loop sequence coincided with the emerging antibody response against this sequence. Additionally, the authors highlighted that the neutralizing activity observed in the samples was partially due to antibodies recognizing the V3 mimotopes.

Besides the multiple reports on the use of RPL to characterize the humoral response against HIV-1 Env proteins, Gupta *et al.* evaluated the reliability of using targeted antigen gene fragment libraries for the identification of epitopes recognized by antibodies elicited in rabbits immunized with p24. To this end, they constructed a phage library composed of DNAse-digested fragments of Gag DNA [58]. Phagotopes obtained after the first panning round displayed mainly 30–40-mer peptides, 70% of which mapped to of the *N*-terminus of p24 (150–240 of Gag) and 30% corresponded to the *C*-terminal region of p24 (310–360 of Gag). Only one phagotope mapped to the central region of Gag (269–310). At the end of the second round, selected phages displaying longer inserts of 40 to 50 AA corresponding to the *N*- and *C*-terminal regions of Gag were identified, revealing the presence of two distinct antigenic regions in Gag. This study demonstrated that gene-fragment phage display could be used to identify epitopes targeted by polyclonal Abs.

### 2.2. Antibodies Directed against Host Proteins

Although they occur at a very low frequency in humans, antibodies targeting host proteins involved in HIV-1 infection have been reported in immunized animals. Given their potential value for viral entry inhibition and the general understanding of this mechanism, RPLs were screened on these MAbs to gain better knowledge of their epitopes (Table 3).

The murine MAbs 3A9 and 5C7 were raised against cells transfected with the seven transmembrane-spanning domains chemokine receptor CCR5, one of the main coreceptors for HIV-1. They recognize a common epitope located near the CCR5 *N*-terminus [67,68]. Both MAbs were used to screen a constrained 9-mer RPL [63]. Phagotopes selected on 3A9 displayed the sequence CHASIYDFGSC while CPHWLRDLRVC was the most prevalent sequence isolated on 5C7. These sequences showed homologies to residues located at the *N*-terminus but also within the first or third extracellular loop (ECL) of CCR5. Both reacted against the targeted MAb either in phage, cyclic peptide or linear peptide formats. Moreover, they were able to bind to gp120 and the peptide selected on 3A9 inhibited binding of the MAb to a cell line expressing CCR5. To further characterize the conformational epitope recognized by 3A9, additional screening rounds of 12-mer, 7-mer and 7-mer-c RPLs were performed [62].

Sequences with an HW motif homologous to the CPHWLRDLRVC motif selected on the MAb 5C7 were identified, and Ala-scanning confirmed the importance of the HW motif and SIYD motifs previously identified for 3A9 binding [63].

Another murine antibody (Mab 2D7) recognizing a conformational epitope on the second ECL of CCR5 [67] was explored by screening a linear 15-mer RPL [64]. Three phagotopes (M14, M23 and M71) were isolated and one of them (M23) was able to inhibit cell infection by the HIV-1 SF162 isolate. The corresponding peptide (FCALDGDFGWLAPAC) fused to the pIII phage coat protein neutralized infection mediated by the JR-FL but not the IIIb strain. The fusion protein specifically bound 2D7 and was recognized in a dose-dependent manner by three CCR5 chemokine ligands, *i.e.*, CCL5 (RANTES), CCL3 (MIP1α) and CCL4 (MIP1ß), confirming its CCR5 mimicry. Six years later, another screening campaign was conducted with a linear 12-mer RPL on 2D7 and the EW**QKEGL**V**TL**WL sequence of a high-affinity binding peptide was obtained [65], revealing that this peptide presented homologies to the *N*-terminal (170-**QKEGL**-174) and *C*-terminal (195-**TL**-196) regions of the CCR5 ECL2. Ala substitutions of the TL residues confirmed their crucial role in 2D7 binding. The selected peptide was used in rabbit immunization studies and elicited Abs with 2D7-like biological functions, *i.e.*, which inhibited HIV-1-mediated cell fusion and PBMC infection.

The CD18 cell surface molecule, a part of the LFA-1 molecule, is involved in the syncytia formation of HIV-1-infected lymphocytes [69]. As MAb MHM23, a CD18 binder, inhibits HIV-1-mediated cell fusion, Poloni *et al.* applied the phage display technology to map the MHM23 epitope and thereby identify the CD18 domains which account for syncytia formation [66]. Linear and constrained 9-mer RPL were panned on the MHM23 MAb, to allow for the selection of linear and constrained sequences. A PPFxYRK consensus motif was inferred by sequence comparison, assigning the epitope recognized by MHM23 to residues 200–206 of CD18. Two phagotopes inhibited *in vitro* HIV-1-induced syncytia formation and one of them retained this ability in the peptide format, confirming its role in syncytia formation and highlighting that mimics of this epitope could prevent cell-mediated viral propagation.

## 3. Identification of HIV-1 Inhibitors by Phage Display

**Figure 4:**
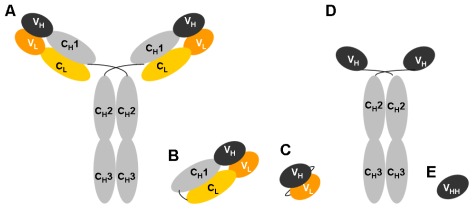
Antibodies and antibody fragments used in phage display. (**A**) Whole human IgG; (**B**) Fab (Fragment antigen binding); (**C**) ScFv (Single-chain variable Fragment); (**D**) Camelid HcAb (Heavy-chain only Antibody); (**E**) V_HH_/Nanobodies (Variable domain of HcAb).

As summarized in the first section of this review, phage-displayed RPLs are powerful tools to determine or to characterize MAbs as well as PAbs epitopes. Besides epitope mapping the phage display technology was also widely applied to the identification of HIV-1 inhibitors. Screening of phage-displayed RPLs, antibody-fragment or ligand libraries on viral or host targets contributed to the discovery of molecules interacting with the key players of HIV-1 infection. Antibody libraries were particularly investigated, and repertoires of Fab, ScFv (antibody fragment corresponding to variable regions of the heavy (VH) and light (VL) chains of antibody connected by a short peptide linker), V_HH_/nanobodies (single domain antibody fragment (SdAb) corresponding to the variable heavy-chain domain of a camelid heavy-chain only antibody (HcAbs)) or CDR3 fragments from naïve or HIV-1 infected subjects as well as from immunized animals were displayed at the surface of phages (Figure 4).

### 3.1. Inhibitors of HIV-1 Proteins

Most of the HIV-1 inhibitors selected with the help of phage display were identified by targeting viral proteins (Table 4).

#### 3.1.1. Env Inhibitors

##### 3.1.1.1. Gp120 CD4 Binding Site

The CD4 binding site represents one of the main Achille’s heels of the virus since it is involved in the earliest step of HIV-1 entry and is conserved in almost all HIV-1 strains [126,127]. Numerous phage display biopannings were performed on gp120 and are classified here according to the type of antibody libraries used.

###### 3.1.1.1.1. Fab Libraries

Burton *et al.* were the first to report the construction of a phage-displayed Fab library from the bone marrow of an asymptomatic HIV-1-infected patient with high titers of gp120-specific Abs [41]. This library was screened against recombinant gp120 from the IIIb isolate and clones displaying high affinities (<10 nM) for gp120 were selected [42]. One Fab (b12) (See Section 2.1.1.4.) was able to neutralize the MN and IIIb strains in different set-ups. This Ab is the most potent neutralizing Ab isolated to date, featuring neutralizing activity against 75% of 36 primary isolates of HIV-1 tested at concentrations that could be achieved by passive immunization [73].

To improve its affinity, the b12 Fab was submitted to CDR walking, a procedure involving randomization of its CDR and expression of the derived libraries expressed on phages, followed by screening against on gp120 [73]. Sequential CDR walking of the HCDR1 and HCDR3 domains was performed and four clones were chosen for detailed analysis of their binding affinity and neutralization potency against the IIIb and MN isolates. A Pro96Glu mutation of the HCDR3 was identified in the clone with highest affinity, 3B3, which bound IIIb gp120 with an 8-fold improved affinity (0.77 nM) compared to the parental b12 Fab.

Similarly, Fab 3B3 was able to neutralize four isolates that were insensitive to the parental b12 Fab. The CDR walking mutagenesis strategy was pursued in a subsequent study and a further 420-fold improvement of the binding affinity of 3B3 for gp120 was achieved, reaching 15 pM [72]. These studies were the first to demonstrate that recombinant Fabs (devoid of the typical IgG contamination residual of calpain cleavage) featured neutralizing activities similar to those of whole IgG. As the Fabs fragments were easier to produce and their smaller size allowed them to target binding sites that were not accessible to full-length Igs, this led to the construction of many Fab libraries to elucidate the immune response to HIV-1 and to identify therapeutic antibodies.

The extremely high binding affinity of 3B3 was also applied to develop an immunotoxin which could specifically kill HIV-1-infected lymphocytes [71]. The authors engineered 3B3 ScFv fused to a truncated form of Pseudomonas exotoxin A. The 3B3(Fv)-PE38 fusion immunotoxin bound to the MN strain of gp120 with the same affinity as the parental Fab antibody and specifically killed a gp120-expressing cell line and a chronically HIV-infected lymphocytic cell line. This study provided the proof-of-concept that high affinity anti-HIV-1 antibodies have a dual application since they may be used for their neutralizing potency but also as carriers for antiviral compounds.

Most antibodies obtained through screening Fab libraries against monomeric gp120 targeted epitopes related to the CD4-binding domain of gp120, pointing to it as an immunodominant epitope [70]. To expedite the identification of NtAbs directed against weakly immunogenic epitopes, a strategy called epitope-masking was applied in several studies. This biopanning approach is designed to mask a particular epitope with antibodies or ligands directed against the region of interest prior to addition of the phage library. Ditzel *et al.* panned a Fab library from an asymptomatic HIV-1-infected patient on immobilized recombinant gp120 and identified two dominant clones targeting the CD4-binding site [74]. These two Fabs were then incubated with gp120 to mask their respective epitopes and the panning of the library was repeated, highlighting (based on sequence similarities) four groups of Fabs recognizing gp120 with affinities in the range of 50 to 100 nM. Epitope mapping of one representative Fab for each group showed that gp120 binding of three clones was influenced by the V2 loop and the CD4-binding site and was not affected by the glycosylation status of gp120. Furthermore, one of these Fabs (L78) featured a broad neutralization spectrum against various HIV-1 strains. The authors performed further epitope masking by using different selection strategies with the same Fab phage library [81]. The first strategy involved masking the CD4-binding site (CD4BS) epitopes either with soluble CD4 or with a CD4BS Ab. All Fabs selected on sCD4-bound gp120 recognized the C1 region, while the Fabs isolated from the CD4-BS Ab-captured gp120 were classified in four different groups: (i) Fabs targeting the C1 region, similarly to Fabs isolated on sCD4-bound gp120; (ii) Fabs directed against the C1–C5- region; (iii) Fabs recognizing the V2 loop; and (iv) Fabs directed against a CD4BS/V2 loop region, similar to the neutralizing FAb isolated by Ditzel *et al.* [74]. Multiple epitope masking was then conducted by masking the CD4BS-MAb-captured gp120 with one of the C1-specific Fabs selected on the sCD4-bound gp120 prior to phage addition, leading to the identification of two C1/C2-dependent Fabs. All isolated Fabs bound their targets with affinities ranging from 4 to 300 nM. However, these FAbs targeting weakly immunogenic regions were not or poorly neutralizing.

More recently, Koefoed *et al.* investigated the anti-gp120 Ab repertoire of the circulating gp120-binding IgG-bearing B cells of 22 HIV-1-infected patients by constructing phage displayed Fabs libraries from unselected cells or from cells preselected with immobilized gp120 [128]. Panning against gp120 selected for a higher number of phagotopes from the preselected library. Clones from the unselected library recognized the V3 loop, while clones from the preselected library targeted the CD4 BS or a CD4-induced epitope encompassing the C1 region. These Fabs displayed no significant differences with respect to epitope specificity, affinity and neutralization ability compared to Fabs obtained from bone marrow libraries, and most of them were unable to neutralize HIV-1. These results were in accordance with previous findings by Parren *et al.* (1997) concluding that the majority of the circulating HIV-1 specific antibodies were elicited by viral debris and were therefore devoid of neutralizing activity [129].

###### 3.1.1.1.2. ScFvs Hydrolyzing gp120

Antibodies recognizing the amino acids 421–436 of the gp120 CD4BS were isolated from patients suffering from the systemic lupus erythematosus autoimmune disease [130,131]. However, whether these antibodies neutralized HIV-1 was not known, which prompted Karle *et al.* to quantify gp120-recognizing Abs in an existing ScFv phage-displayed library from the PBMCs of lupus-suffering patients [132]. Biopanning selected for clones binding both gp120 and the 421–436 region of the gp120-CD4-binding site. One of these clones (JL413) neutralized R5 and X4-tropic HIV-1 primary isolates from clades B, C and D with IC_50_ ranging from 0.1 to 25.6 μg/mL.

A subset of gp120-binding antibodies was shown to hydrolyze gp120 by a mechanism analogous to serine protease [133], As the nucleophilic region responsible for this activity was localized in the light chain [134,135], a library of light chains prepared from three lupus patients [132] was screened with an electrophilic analogue of gp120 residues 421–433 to isolate antibodies capable of binding and hydrolyzing gp120 [75]. One of the light chain clones selected (SKL6) cleaved a gp120 421–433-reporter substrate as well as full-length gp120. Engineering of Abs composed of such a light chain coupled to a gp120-binding heavy chain might provide Abs with anti-viral proteolytic activities.

###### 3.1.1.1.3. Nanobodies

The advantages of reduced antibody formats were also explored with naturally occurring smaller antibodies. In addition to conventional antibodies, camelids also produce antibodies devoid of light chains (Figure 3D). These heavy-chain only antibodies (HcAbs) lack the C_H1_ domain and their binding specificity is provided by the variable heavy-chain domains of HcAbs (V_HH_ or nanobodies) [136]. The CDR regions of nanobodies are on average longer CDR than those of conventional Abs and display a protruding conformation, thereby more easily binding clefts and active sites. Nanobodies were successfully used for panning against various pathogens (reviewed by Vanlandschoot [137]). Forsman *et al.* immunized llamas with a recombinant gp120 of subtype B/C (CN54) [78]. Three different panning strategies against various gp120 resulted in the selection of three nanobodies (A12, D7 and C8) with neutralizing activity against a limited panel of clade B and C strains (IC_50_ values ranging from 0.003 to 38 μg/mL). These nanobodies bound gp120 with affinities from 0.1 to 1 nM and inhibited its binding to CD4 as well as to MAbs known to recognize the gp120 CD4 binding site and, last but not least, competed with each other for gp120 binding. In a follow-up study, Koh *et al.* described a family-based approach to produce nanobodies similar to A12 and D7 [77]. They used a degenerated oligonucleotide annealing to the last six codons of CDR3 and framework (FR) 4 and an FR1-specific primer to amplify a sublibrary of related V_HH_ clones with properties similar to the parental A12 or D7 V_HH_. More than 30 phagotopes were tested for their ability to neutralize 3 clade B and 3 clade C strains. All tested V_HH_ displayed similar neutralizing activity against the clade B viruses. Three different neutralization profiles (Broad A12-like, Intermediate and Narrow D7-like potency) were observed against the clade C type strains and the breadth of neutralization potency appeared to be correlated to the presence of an YYD motif in the *C* terminus of A12 CDR3. When the CDR3 YDD motif from A12 was introduced within D7, gp120 binding affinity increased by 10-fold and neutralization of clade C strains increased by 5-fold. These studies were the first to demonstrate BNtAbs elicited in immunized animals. As V_HH_ are stable and can be produced at a relatively low cost, they are promising HIV-1 inhibitors.

###### 3.1.1.1.4. CD4 Mimics

The CD4 receptor recognizes gp120 through residues located within its V1 region, and engineering of this cell receptor was applied to identify CD4 variants with a better affinity for gp120, thereby displaying HIV-1 inhibitory properties. In 1997, Krykbaev *et al.* constructed a phage-displayed library of CD4 V1 and V1-V2 variants generated by error-prone PCR and screened it against gp120 [79]. Five clones with increased affinity for gp120 and presenting mutations within the CD4 V1 domain were identified. All of these clones inhibited HIV-1 entry with IC_50_ ranging from 0.2 to 1 μg/mL.

##### 3.1.1.2. Gp120 V3 Loop

The phage display technology was used to improve the affinity of the Mab 447-52D for its V3 loop epitope, which had also been identified through phage display [16,17]. In 1996, Thompson *et al.* expressed MAb 447-52D as ScFv on phages and combined its V_H_ with λ and κ chains from a non-immunized PBL repertoire prior to panning on a peptide containing the V3 loop sequence [80]. Additional shuffling of the HCDR1 and HCDR2 regions was combined to HCDR3 “spiking”, *i.e.*, the introduction of random mutations, resulting in the identification of four key residues that could be mutated to improve Ab affinity. A sublibrary in which all four codons were simultaneously mutated was constructed and biopanning allowed to select for one ScFv (402P5H7) with improved K_D_ against the MAb 447-52D epitope. Two neutralizing Fabs (Fab loop 2 and Fab DO142-10) were obtained by screening a Fab library against recombinant gp120 reaching IC_50_ ranging from 0.2 to 8 μg/mL [70,81].

##### 3.1.1.3. Gp120 CD4-Induced Epitope

In 1999, Ferrer and Harrison screened 7-mer, 12-mer and constrained 9-mer RPL against gp120 and identified two peptides from the 12-mer RPL [82]. The first sequence RINNIPWSEAMM (12p1) inhibited CD4 as well as NtMAb17b binding while the second peptide, TSPYEDWQTYLM (12p2) did not affect the CD4 interaction and rather enhanced 17b binding. The 12p1 peptide was further investigated and shown to inhibit binding of monomeric YU2 gp120 to both sCD4 and 17b with IC_50_ values of 1.1 and 1.6 μM respectively. The 12p1 peptide also inhibited binding of these ligands to trimeric envelope glycoproteins, blocked binding of gp120 to the native coreceptor CCR5, and specifically inhibited HIV-1 infection of target cells *in vitro* [138].

HIV-1 entry is a multi-step process requiring the successive binding of gp120 to CD4 and to a coreceptor, CCR5 or CXCR4, and triggers successive conformational changes that expose transient epitopes. Targeting of these epitopes with NtAbs could therefore prevent HIV-1 infection, as has been proven with the clinically approved fusion inhibitor Enfuvirtide. To identify such receptor-induced epitopes, Moulard *et al.* screened a Fab library constructed from an HIV-1 infected patient (FDA-2) with high NtAb titers against gp120-CD4-CCR5 complexes [83]. One Fab clone, X5, bound gp120 from several strains with a low nanomolar affinity. Furthermore, binding affinity was significantly increased in the presence of CD4 and slightly enhanced by CCR5. Competition assays with a panel of antibodies targeting different HIV-1 epitopes revealed that X5 recognized an epitope located in close vicinity to the CD4 and coreceptor binding sites. Neutralization assays with isolates from clades A, B, C, D, F and G demonstrated that X5 neutralized all isolates with potency comparable to that of b12. X5 is the first BNtAb recognizing a receptor-induced epitope identified to date. To select for ligands for CD4-induced epitopes, murine leukemia virus particles carrying the Env protein of the dual-tropic 89.6 strain pre-incubated with sCD4 were recently used to screen 7-mer, 7-mer-c and 12-mer RPLs [84]. One of the selected phagotopes (XD3: HKQ**P**W**YD**YWLLR) displaying sequence similarities (in bold) with the *N*-terminal extracellular part of the CCR5 and CXCR4 coreceptors was identified, suggesting novel potential leads for tyrosine sulfation. Both the XD3 phagotope and XD3 peptides strongly and specifically bound to 89.6 gp120 regardless of CD4 and XD3 competed with MAb 17b for binding to a CD4-induced epitope. The sulfated form of the XD3 peptide recognized X5, R4 and dual-tropic strains and inhibited HIV-1 entry in the high micromolar range.

##### 3.1.1.4. Gp120 C1 Domain

The Salp15 salivary protein of *Ixodes scapularis* inhibits CD4+ T cells activation by binding to the CD4 molecule in a region that may overlap with the gp120 binding site. This inhibition is mediated by the *C*-terminal 95–114 GPNGQTCAEKNKCVGHIPGC sequence [139]. Juncadella *et al.* thus analyzed Salp15 as a potential HIV-1 inhibitor and demonstrated that Salp15 inhibited gp120-CD4 interaction and subsequent cell fusion and that the GPNGQTCAEKNKCVGHIPGC peptide also interacted with gp120 [140]. To identify which gp120 amino acids interacted with peptide 95–114 of Salp15, the authors screened a 7-mer RPL against Salp15 and isolated a HVITPLW sequence homologous to an (I/L)TPL motif of the gp120 C1/V1 domain which is highly conserved across HIV-1 isolates. Finally, they mapped the interaction site of full-length Salp15 protein or of its 95–114 *C*-terminal domain were able to bind to the PCVK**LTPL**CVTLNCT peptide within the gp120 C1/V1 region.

##### 3.1.1.5. Phage Display as a Tool to Unravel the HIV-1-Specific Humoral Response

In addition to epitope mapping and inhibitor identification, phage display was also widely applied to elucidating the determinants of the initial response to HIV-1 antigens and more particularly the importance and different roles of IgM and IgG during the establishment of infection. Indeed, all known BNtAbs are IgGs that are somatically hypermutated and are thus more difficult to elicit. In contrast, IgMs are closer to germline antibodies and the identification of HIV-1 specific IgM could be relevant for the development of vaccine immunogens. The studies listed in this section explored and emphasized the importance of the initial IgM response against HIV-1 and viral strategies to skew it towards non-neutralizing or infection-enhancing antibodies.

Screening against gp120 with IgM and IgG Fab repertoires constructed from a healthy donor demonstrated that only Fabs isolated from IgM were able to recognize gp120, although they were polyreactive, displayed low affinities and no neutralizing properties [141]. Sequence analysis evidenced that selected gp120-binding Fabs originated from different V_H_ germline genes. Several studies reported that gp120 displays superantigenic properties giving it the ability to bind and stimulate non-immune B cells to secrete V_H_3 Ig *in vitro* [142]. Interestingly, the V_H_3 antibody family is the most represented immunoglobulin gene family in healthy adults (54% of peripheral repertoire) and HIV-1 infection leads to altered V_H_3 production through selective depletion of the anti-HIV-1 V_H_3 antibodies [143]. Toran *et al.* further applied the phage display technology to examine and compare the human V_H_3 genes involved in IgM and IgG responses to gp120 to identify the correlates of long-term non progression. Two IgM and IgG phage-displayed Fab libraries from an HIV-1-infected LTNP with high gp120-specific IgM and IgG1 titers were constructed and screened [144]. Several clones were selected from the IgM library (M02, M025, 4M26 and 4M40) and three clones from the IgG library (S20, S19 and S8). All IgM Fabs were polyreactive and had a binding affinity for gp120 in the micromolar range while the IgG Fabs were specific and bound gp120 with affinities in the nanomolar range, as expected. Sequence analysis showed that IgG Fabs originated from the same germline Ab. The IgM Fab M025 displayed the same V_H_ region nucleotide substitutions as those of IgG Fab S8 and used similar D_H_ and J_H_ segments, suggesting that S8 arose from M025 by isotype switching. In addition, a four aminoacid difference in the HCDR3 sequence of M025 (TGQWE) and S8 (RGGSI) was proposed to be associated with the 100-fold affinity increase for gp120 and to the higher neutralizing activity of IgG Fab S8 (ID_50_ = 23 ng/mL) than of IgM M025 (ID_50_ = 3 μg/mL). In a follow-up study conducted two years later, the three IgG Fabs were submitted to reverse mutations to reconstitute the germline amino acid residues [145]. The higher affinity and neutralizing ability of S20 were due to the Ala30Arg and Ala31Asp somatic mutations in the HCDR1 region of the germline gene sequence, providing clues for rational modifications of CDR in human antibodies to improve affinity and HIV-1 neutralization capacity.

The IgM to IgG isotypic switch generating high affinity neutralizing or non-neutralizing antibodies is triggered by the activation of IgM-producing B cells. To characterize the epitopes recognized by HIV-1-specific IgM and to assess the effects of these Abs on HIV-1 infection, Chen *et al.* constructed a Fab library from blood, lymph nodes and spleen from 59 healthy donors [146]. The library was panned against gp140 (Env ectodomain containing both gp120 and a truncated gp41 lacking transmembrane domain and cytoplasmic tail) of a clade B isolate and allowed for the selection of one Fab clone (R3H1m) with a relatively high binding affinity for gp140 from different strains. A sublibrary derived from this clone was panned against gp140 from different isolates, resulting in the selection of clones (m19, m19a, m19b, m19c and m19d) binding with high affinity to clade B and F gp140 (EC_50_ ranging from 2 nM to 80 nM). While these antibodies had weak neutralizing properties against X4-tropic isolates, they did not inhibit and in some cases even enhanced infection with R5-tropic isolates. The m19 Ab, whose sequence is relatively similar to the germline Ab, targeted highly conserved epitopes located near the CD4 binding site or the coreceptor binding site. The high immunogenic capacity of the conserved non-neutralizing epitopes of such antibodies could divert the immune system from actually neutralizing epitopes. The authors suggested that these newly identified MAbs could be used as probes to further characterize conserved non-neutralizing or enhancing epitopes and to modify or remove them from candidate vaccine immunogens. The epitope masking strategy (section 3.1.1.1.) might be applied to these epitopes to redirect the immune system to elicitation of antibodies targeting neutralizing epitopes.

To select for antibodies closely resembling the germline antibodies as a candidate source of BNtAbs, the same authors constructed a cord-blood-derived IgM library which they submitted to parallel screening against HIV-1 Env, SARS coronavirus protein binding domain (RBD) and soluble Hendra virus G protein (sG) [147]. Although RBD and sG antigens provided enriched IgM, the library could not be enriched in HIV Env-binding phagotopes. These results are in accordance with the hypothesis that HIV-1 could have evolved strategies based on weak or absent binding to antibodies of the germline repertoire [146]. Presenting epitopes unsuitable for binding of somatically hypermutated antibodies would enable the virus to escape from strong immune responses.

##### 3.1.1.6. Gp41 MPER Inhibitors

In 2001, Zwick *et al.* constructed a phage-displayed Fab library from an HIV-1 positive patient with exceptionally BNtAbs (FDA-2 patient). This library was screened on the MN2031 peptide encompassing the 2F5 epitope ELDKWA, and led to the identification of the Z13 MAb [85]. Epitope mapping experiments based on synthetic peptides and recombinant proteins showed that the epitope targeted by MAb Z13 was located downstream of the 2F5 epitope and centered on the NFWDIT sequence. MAb Z13 neutralized primary isolates from HIV-1 B, C and E subtypes. To enhance binding potency, Fabs sublibraries of Z13 variants were engineered and screened against an MPER peptide and gp41 [86]. The selected Z13e1 variant displayed an over 100-fold increase in neutralization breadth and potency compared to the parental Z13 MAb. Binding experiments coupled to competition assays revealed that the Z13e1 MAb bound to a WASLWNWFDITN minimal epitope overlapping the 2F5 epitope and that the Asn and Asp residues were essential for peptide recognition as well as HIV-1 neutralization.

Very recently, phage- and yeast-displayed Abs libraries constructed from an HIV-1-infected patient with 2F5-like BNtAbs were panned against peptides containing the 2F5 epitope and against the HIV-1 JR-FL gp140 [148]. Two MAbs (M66 and M66.6) were identified and the most mutated variant (M66.6) neutralized HIV-1 with a higher potency than M66. Ala substitutions indicated that both Abs recognized the DKW core of the 2F5 epitope and two additional Leucine residues located upstream (L(660,663)).

##### 3.1.1.7. Gp41 Heptad Repeat Inhibitors

Fusion of viral and host membranes, the last step of HIV-1 entry, requires the initiated by the insertion of the gp41-encoded fusion peptide into the host cell membrane and the formation of an extended prehairpin intermediate (PHI). The gp41 *N*- and *C*-terminal heptad repeats (NHR, CHR) then collapse to form a six-helix bundle (6-HB) in which the NHR form a trimeric coiled-coil, creating grooves where the CHR bind. The viral and cellular membranes are thereby brought into close proximity, enabling fusion (Figure 1). During PHI formation, the NHR and CHR do not interact and may thus be transiently targeted by compounds which prevent the formation of the six-helix bundle in a dominant-negative manner. One such compound is the synthetic CHR mimic Enfuvirtide (T20) [4,149]. A hydrophobic pocket located on the *N*-terminal peptide trimer groove of the 6-HB is highly conserved among HIV-1 sequences and plays a critical role in membrane fusion, and therefore represents a select target for inhibitors.

Eckert *et al.* used the particular approach called “mirror-image phage display” to identify d-peptides targeting the gp41 hydrophobic pocket [89]. In this approach, a phage library of natural l peptides is screened against the mirror image of a target synthesized as d-peptide [150]. By symmetry, the selected d-peptide phagotope sequences will bind the natural l-form of the target. The main advantage of d-peptides over l-peptide inhibitors is their resistance to natural proteases which enhances their oral bioavailability and serum half-life. In a first study, the authors screened a constrained 10-mer RPL against the d-peptide sequence of the gp41 hydrophobic pocket fused to a soluble trimeric coiled-coil (IZN17). Pocket-specific binders with a consensus motif Cx_5_EWxWLC were identified and inhibited cell fusion or HIV-1 entry into cells with an IC_50_ in the micromolar range when synthesized using d-amino acids. In a second study, the same authors constructed a sublibrary based on the consensus sequence identified in their first study [89] which allowed the selection of sequences with a fourfold potency increase [87]. Surprisingly, the most potent peptide was a 8-mer with a Cx_3_EWxWLC motif which was probably selected from the 10-mer sublibrary because its smaller size favored a more compact hydrophobic core upon binding to the gp41 hydrophobic pocket. Screening of second generation d-peptides from a 8-mer CX_4_WXWLC library led to the selection of pocket-specific inhibitor of entry (PIE) 7 with an IC_50_ of 620 nM. Dimeric and trimeric forms of PIE7 had respective IC_50_ values of 1.9 nM and 250 pM. In a third study, the same authors constructed a phage library based on the PIE7 core sequence flanked by two randomized amino acids (xxCDYPEWQWLCxx) and obtained phages with the H(A/P)-[PIE7 core]-(R/K/E)L consensus sequence [88]. A peptide (PIE12, HPC**DYPEWQWL**CEL) exhibited a broad neutralizing spectrum and was even more efficient than T20, reaching an IC_50_ of 0.5 nM, when trimerized. Moreover, this third generation PIE12-trimer displays broadened inhibitory potency and resistance to viral variants, as escape mutants required over 65 weeks of selection *in vitro* to emerge. The PIE12 trimer is thus a promising entry inhibitor and may be used as a topical microbicide in its D conformation.

In 2005, there was no evidence of Abs capable of binding the highly conserved NHR region targeted by the T20 inhibitor. To determine whether antibody fragments could target this determinant on the gp41 protein, Miller *et al.* constructed a phage-displayed naïve ScFv library and screened it against a synthetic protein mimicking the 6-HB [90]. This construct, named 5-Helix, lacks one of the three CHRs and the NHR trimer is partially exposed, presenting a single binding site for a CHR mimic [151]. The ScFv library was also panned against the IZN36 compound, a homotrimerized form of 36 NHR amino acids fused to a coiled-coil peptide, therefore representing a 6-HB mimic devoid of the CHR trimer [91]. The authors identified a ScFv (D5), which blocks HIV-1 entry and inhibits infection in a single-cycle infectivity assay. This ScFv retained its properties when produced as a whole IgG1. The antibody was found to bind the hydrophobic pocket of the NHR trimer and Ala scan experiments revealed the crucial role of residues L568, W571, and K574 located in the hydrophobic pocket for this interaction. IgG1 D5 was able to neutralize at least five HIV-1 isolates with IC_50_ ranging from 93 to 1750 nM, thereby demonstrating that the hydrophobic pocket of the NHR trimer is accessible for binding of HIV-1 inhibitors as large as IgGs.

The same year, another study demonstrated that antibodies binding with high affinity and specificity to heptad repeats can be isolated from synthetic Fab minimalist libraries presenting Tyr/Ser randomization of their CDR [152]. Very recently, Liu *et al.* took advantage of these results and constructed a minimalist Fab library where the LCDR3 and HCDR1-3 domains were randomized with Tyr and Ser [92]. Screening of this library on the 5-Helix mimic selected for Fabs with affinity and specificity values comparable to those obtained with the ScFv of the previously described D5 Ab [90,153]. Huang *et al.* used the N34(L6)C28 polypeptide mimicking the 6-HB [154] to screen 7-mer and 12-mer RPLs and selected sequences bearing a Hxx(N/D)PF motif [93]. This consensus motif synthesized as peptide (JCH-4) inhibited HIV-1-mediated syncytia formation [94].

Fusion inhibitors were also identified by screening non-immune human Fab libraries. Louis *et al.* screened such a library [155] against antigens comprising the trimeric coiled-coil NHR fused or not to the gp41 six-helix bundle (N35CCG-N13 and NCCG-gp41, respectively) [156,157]. They identified Fabs targeting (i) the 6-HB; (ii) the NHR trimeric coiled-coil or (iii) both 6-HB and trimeric coiled-coil [95]. These antibodies were tested in a cell fusion inhibition assay and the two more potent MAbs, belonging to the third group, featured an IC_50_ of 6–7 μg/mL. Two years later, the same library was screened against NCCG-gp41 and 6-HB antigens [96]. Two clones, Fabs 3663 and 3670, inhibited cell fusion while one Fab 3674 clone selected against NCCG-gp41 was also effective in infection neutralization assays. Fab 3674 bound the 6-HB as well as stable NHR trimers, and recognized an epitope that partially overlapped the hydrophobic pocket targeted by the D5 Ab. The same authors subsequently demonstrated that the N36^Mut(e,g)^ peptide presenting mutations within the 5^th^ and 7^th^ AA residues of the heptad repeat [158] increased the temporal window of viral sensitivity to Fab 3674 and thereby synergistically enhanced the neutralizing activity of Fab 3674 as well as of the BNtAbs 2F5 and 4E10 [98]. A Fab sublibrary was created by affinity maturation of the Fab 3674 HCDR2 loop and screened against NCCG-gp41, selecting for three Fabs (Fabs 8060, 8066 and 8068) with enhanced potency (average 5-fold decrease in IC_50_) and neutralization breadth [97].

To follow-up a study demonstrating that affinity-purified IgGs from rabbits immunized with N35_CCG_N13 inhibited HIV-1-mediated fusion [156], Nelson *et al.* rescued a ScFv antibody library from these animals [99]. Three N35_CCG_N13 binders were selected, and one of them, 8K8, displayed neutralizing activity against HXB2. In parallel a more complex Fab library was constructed from the FDA-2 HIV-1- positive patient from whom Z13 Ab had previously been isolated [85]. Screening this library against N35_CCG_N13 allowed for the isolation of Fab DN9 [99]. Both ScFv 8K8 and Fab DN9 neutralized HIV-1 infection with a panel of viral strains with IC_50_ ranging from 50 to 500 nM and targeted the NHR trimeric coiled-coil, presumably close to the hydrophobic pocket. Three additional gp41-specific Abs (M44, M46 and M48) were obtained by screening antibody phage libraries from asymptomatic seropositive patients [159] against gp140 [100–102]. A recombinant gp140 (gp140 R2) isolated from an asymptomatic seropositive patient with BNtAbs was reported to elicit BNtAbs in monkeys, further demonstrating that immunogenic epitopes were exposed on this recombinant antigen [101]. Competitive antigen panning (CAP) (biopanning approach designed to outcompete phagotopes binding to an immunodominant region of a multi-domain target through concomitant addition of an excess of soluble forms of this immunodominant domain) using a mixture of gp140R2 as antigen and gp120R2 as competitor resulted in the selection of a gp41-specific M46 Ab [101]. M46 displayed broad neutralization properties and recognized a conformational epitope and bound weakly to 5-Helix antigen but not to the trimeric NHR nor to 6-HB. In two other studies, the same libraries were panned against gp140/120 from three different isolates (89.6, cm243 and R2), which led to the identification of the M48 Ab recognizing a conformational epitope of gp140 [102] and the M44 Ab, which binds gp140, 5-Helix and 6-HB but not to the NHR trimeric coiled-coil. M44 recognized a conserved conformational epitope and neutralized isolates from different clades with a significantly higher potency than 4E10 or Z13 [100]. The competitive antigen panning approach against gp140/120 thus allows the selection of Abs recognizing conformational epitopes on gp41, which are not properly folded when gp41 is used as a target.

#### 3.1.2. Other HIV-1 Proteins

Most of the studies found in the literature that apply the phage display technology to the discovery of HIV-1 inhibitors target the Env protein. However, reports about the identification of peptides directed against other HIV-1 proteins involved in viral replication as well as interfering with RNA sequences have been published and are summarized in this section.

##### 3.1.2.1. Viral Protein of Regulation (Vpr)

Vpr is involved in the nuclear import of the viral preintegration complex (PIC) as well as in the induction of apoptosis after cell cycle arrest and can be packaged into virions in quantities similar to the structural proteins [160–163]. Vpr was also reported to be associated with numerous cellular proteins such as glucocorticoid receptors, transcription factors or the uracyl DNA glycosylase (UDG) [160,164–166].

To investigate whether Vpr may be used as a docking protein to deliver anti-viral compounds into virions, BouHamdan *et al.* used a 7-mer RPL to determine a common motif involved in the interaction between Vpr and its various ligands [103]. Screenings rounds against Vpr fusion proteins pinpointed sequences sharing a WxxF motif. Since UDG contains a WxxF sequence, mutants were constructed and confirmed the importance of this motif for Vpr binding. Cotransfection experiments indicated that the WxxF motif might be used to deliver a fusion protein into the HIV-1 virion through a new docking strategy.

In 2003, Krichevsky *et al.* conducted a study to elucidate the exact role of Vpr and its contribution to the nuclear import process of the HIV-1 PIC [104]. To that aim, a semi-synthetic ScFv library [167] was screened against the *N*-terminal (AA 17–34) part of Vpr (VprN) conjugated to BSA (VprN-BSA). Purified ScFvs fragments featuring their strong and specific binding to the VprN sequence recognized full-length Vpr and inhibited Vpr-mediated nuclear import, indicating that targeting Vpr may lead to the development of new peptides to fight viral infection.

##### 3.1.2.2. Integrase (IN)

The phage display technology has also been applied to the identification of the HIV-1 integrase inhibitors. In 2004, Desjobert *et al.* screened a 7-mer RPL against recombinant HIV-1 integrase and identified a high affinity phagotope displaying the FHNHGKQ sequence [105]. In peptide format, this sequence inhibited the strand transfer activity of IN by competing with the target DNA, providing the proof-of-concept that IN is also a valuable target for phage display.

##### 3.1.2.3. Transactivator of Transcription (Tat)/Transactivation Response element (TAR)

Interaction of the viral transcription activator Tat with the human cyclinT1 subunit of the positive transcription elongation factor (P-TEFb) complex and the cooperative binding of this complex to the transactivation response element (TAR) RNA are prerequisites of HIV-1 transcription [168]. Screening RPLs or Fab libraries against Tat, cyclinT1 or TAR elements using the phage display technology identified peptides impairing Tat-mediated HIV-1 replication.

The first study was conducted in 1996, when Pilkington *et al*. screened a Fab library constructed from the Ab repertoire of an HIV-1-infected asymptomatic patient and selected Fabs recognizing a region comprised between amino acids 22 to 33 of the Tat protein in a conformation-dependent manner [106].

Many years later, a non-immune human ScFv phage-displayed library was explored to identify peptides binding to cyclinT1 [107]. Clones recognizing the cyclin box domain of cyclinT1 or interacting with the Tat/TAR recognition motif (TRM) were isolated after panning against the 272 *N*-terminal amino acids of cyclinT1. When expressed as intrabodies (antibody or antibody fragment expressed intracellularly), one of these ScFvs inhibited Tat-mediated transactivation without impairing cellular basal transcription or inducing apoptosis and partially inhibited HIV-1 replication in cultured cells.

The TAR RNA sequence adopts a specific structure recognized by the basic Arginine Rich Motif (ARM) of Tat and thus represents a potential target for phage display screening. In 2005, Kolb and Boiziau screened a 12-mer RPL against TAR RNA molecules and selected 12-mer and unexpectedly 57-mer sequences from the library [108]. The latter were proposed to arise from incomplete enzyme restriction during the construction of the initial library. Clones were further characterized in peptide format and displayed TAR-specific binding. The authors suggested that the surprisingly long peptides might have been selectively retrieved from the library because they presented a conformation that shorter 12-mer peptides were unable to adopt.

##### 3.1.2.4. Nucleocapsid (NC)/Packaging Signal (psi) Sequence

The HIV-1 nucleocapsid protein p7 (NCp7) is processed from the Gag precursor and is involved in the protection and encapsidation of viral RNA leading to viral assembly through interaction with a specific secondary structure of the 125-base long psi RNA [169–172]. Lener *et al.* screened a constrained 9-mer RPL against NCp7 and selected phagotopes sharing a PPx(D/E)R consensus motif [109]. Further binding experiments suggested that the NCp7-phage interactions involved amino acids 30 to 52 of NCp7, encompassing a zinc finger domain.

Studies to identify inhibitors of viral packaging were also conducted. RPLs were screened on the psi RNA immobilized onto a streptavidin-coated surface by annealing its 5′-end to a biotinylated oligomer, leading to the selection of peptides characterized by an HWWPWW motif [110]. Peptide variants presenting this motif were subsequently synthesized and the most efficient binder was shown to strongly reduce virus release by infected cells, suggesting that it could serve as a lead compound to develop new anti-HIV-1 drugs [111].

A similar screening campaign was conducted, where the 5′-end of the psi RNA was covalently immobilized, leaving the secondary structure intact and fully accessible [112]. Screening of a 12-mer RPL selected for four clones with either WHxT or HSSxY motifs which were assessed for specific and dose-dependent binding to psi RNA. The most prevalent sequence (SYQWW**WH**SPQ**T**L) was expressed in fusion with the maltose-binding protein and was able to compete with NCp7 for binding to psi RNA, confirming the value of the peptide as a potential HIV-1 inhibiting compound.

##### 3.1.2.5. Negative Factor (Nef)/Virion Infectivity Factor (Vif)

HIV-1 accessory proteins Nef and Vif have an important role in HIV-1 viral replication and infectivity and, as such, represent as such interesting targets for inhibitors. In 2001, Yang *et al.* demonstrated that Vif was able to multimerize and that its 151-AALIKPKQIKPPLP-164 domain was critical for multimerization pointing to it as an interesting target to impair Vif-mediated viral replication [116]. The authors therefore, screened a 12-mer RPL library against Vif and selected phages sharing a common PxP motif [117]. Four of these sequences synthesized as peptides bound the *C*-terminus of Vif with high affinity and were able to inhibit Vif-Vif as well as Vif-Hck tyrosine kinase interactions. Moreover, these peptides inhibited HIV-1 replication in cultured cells.

Besides the identification of inhibitors from RPL, the discovery of Abs targeting Nef and Vif applicable to intrabody-based therapy may represent an alternative way to impede viral replication [72]. In a very recent study, Yoshikawa *et al.* evaluated the effect of two different schedules of Nef and Vif administration for mice immunization prior to the construction of ScFvs phage-displayed libraries [113]. Results demonstrated that the immunization protocol influenced the complexity of the elicited Ab repertoire and thus the successful identification of Abs specifically recognizing the target.

A nanobody (sdAb19) recognizing a conformational epitope and reacting with a high affinity (K_D_: 2 nM) with Nef proteins from a panel of HIV-1 M, N, O and P groups was isolated through phage displaying the V_HH_ repertoire of a llama immunized with a purified recombinant Nef protein (fragment 57–205) [114]. When expressed as an intrabody, this anti-Nef sdAb inhibited important biologic functions of Nef both *in vitro* and *in vivo* in CD4C/HIV-1Nef transgenic mice.

The first step in cytotoxic T lymphocytes (CTL) activation is the recognition by a T cell receptor (TCR) of the antigen-derived peptide/MHC class I complex (pMHC). To date, few studies were undertaken to examine antigen presentation at a cellular and molecular level. Nunoya *et al.* screened pooled ScFv libraries [173] on an immunodominant HLA-A*2402(A24) restricted CTL epitope within the Nef protein (Nef138-10; RYPLTFGWCF) [115]. The panning procedure yielded clones binding specifically to Nef 138-10/A24. Clones ScFv3 and ScFv27 were able to bind to Nef 138-10/A24 expressed at the cell surface and retained this specificity when expressed as reconstituted whole IgGs. This recent study was the first to address the identification of monoclonal antibodies binding specifically to an immunodominant HIV-1 CTL epitope loaded on an HLA class I molecule.

##### 3.1.2.6. Reverse Transcriptase (RT)

Reverse transcriptase is a valuable target for anti-HIV-1 compounds, as illustrated by the success of the multiple small compounds used in HAART. In 1996, Gargano *et al.* panned a phage-displayed library of synthetic combinatorial human Fab fragments against recombinant HIV-1 RT [174]. Two Ab fragments that specifically inhibited the RNA-dependent DNA polymerase (RDDP) activity of RT were identified. Both fragments also inhibited the activities of avian and murine retroviral RTs as well as the human DNA polymerase α and prokaryotic DNA polymerases. Because of their lack of specificity, these Abs fragments were not exploited further as anti-HIV-1 molecules.

To develop a panel of recombinant MAbs reacting with different epitopes of the RT, Ohba *et al.* immunized mice with recombinant RT expressed in a vaccinia virus vector and constructed a phage-displayed mice Fab fragments library [118]. Biopanning against recombinant RT led to the identification of two Fab fragments (5F and 5G) able to strongly inhibit the RDDP activity of HIV-1 RT. Epitope mapping and competitive ELISA showed that 5F and 5G recognized an epitope similar or closely related to the epitope targeted by the mouse MAb (7C4) previously described by the same authors [119].

Two years later, a semisynthetic phage display library of human ScFvs with randomized heavy and light chain CDR3 was screened against recombinant RT [120]. Five different ScFv Abs directed against RT were isolated, of which three (F-6, 6E9, 5B11) inhibited the RDDP activity of RT; of note, (F-6) also inhibited RT DNA-dependent DNA polymerase (DDDP) activity. Synthesis of the peptides corresponding to the CDR3 regions of the heavy and light chains showed that the heavy chain CDR3 inhibited RDDP activity while the light chain peptide had no effect. These HCDR3 peptides represent the smallest antibody fragments inhibiting the RT identified to date and demonstrated that HCDR3 repertoire is a potential source of bioactive molecules (see Section 3.2.1.2.).

##### 3.1.2.7. Regulator of Virion Expression (Rev)

Rev is a key regulatory protein. Oligomerized Rev binds to unspliced or singly spliced viral mRNA and ensures its transport to the cytoplasm, thereby allowing the translation of viral gene products. Despite considerable efforts, the structure of Rev is poorly characterized since Rev is refractory to crystallization, mainly because of its tendency to form insoluble aggregates [175]. In the absence of structural information, the phage display technology was used by different authors to map the domains involved in the interaction of Rev with its network of partners. Pilkington *et al.* identified two Rev-specific Fabs from a Fab library derived from the Ab repertoire of an HIV-1-infected asymptomatic patient [106]. These Fabs were directed against sites adjacent to the Rev basic nuclear localization signal (NLS) (residues 52–64) and to the activation domain (residues 75–88). Two years later, Jensen *et al.* screened a 15-mer RPL to identify potential Rev peptidic antagonists [121]. Three groups of sequences sharing a SRLxG(x)_2–3_R motif (group I), sharing a RVV(x)_2–4_RG/A motif (group II) or featuring no sequence similarity (group III), were obtained. Three clones were selected based on their high frequency of occurrence (p1 and p3, group I) or on their strong binding affinity for Rev (p19, group III). They were synthesized as peptides and were shown to retain Rev binding specificity. More recently, llama nanobody libraries from animals immunized with recombinant Rev allowed the identification of 12 Rev-binding nanobodies [123]. One of them (Nb190) prevented or disrupted Rev multimerization by interacting with Lys20 and Tyr23 of the Rev *N*-terminal α-helix [122]. Besides inhibitor discovery, Fabs were recently proposed as “crystal chaperones” to support crystallization of their partners by locking them in specific conformations and blocking aggregation [124]. Stahl *et al.* described the preparation, characterization, and crystallization of an equimolar complex formed between Rev and a chimeric rabbit/human Fab (SJS-R1) selected through phage display [124]. The Rev/SJS-R1 Fab complex was successfully crystallized and the Fab SJS-R1 was shown to recognize a conformational epitope in the *N*-terminal half of Rev. Structural characterization of the crystallized Fab/Rev complex is ongoing and a corresponding ScFv has been engineered and may have anti-HIV-1 properties.

##### 3.1.2.8. Group-Specific Antigen (Gag)

To identify peptides interfering with HIV-1 capsid assembly, Sticht *et al.* screened a 12-mer RPL against the capsid (CA) protein generated by the proteolysis of the Gag precursor and identified phagotopes whose sequences could be classified in four groups [125]. One of these sequences (CAI, capsid assembly inhibitor) competed with phagotopes for binding to CA and inhibiting capsid assembly *in vitro*. Interaction with CAI was mapped to CA amino acids 162–190, with additional contacts in helix 4. CAI did not inhibit capsid assembly *in vivo*, but may nevertheless serve as tool for drug screening and as a starting point for drug design based on its CA-binding properties.

#### 3.1.3. Diagnostic Applications

Peptides and antibody fragments selected by means of phage display may also be used for diagnostic purposes or to assess the diversity of the immune response against HIV-1-specific antigens. De Haard *et al.* constructed a ScFv library from PBLs of an HIV-1 positive patient presenting antibodies against gp120, gp41 and p24 and screened the library against gp160 and p24 [176]. One phagotope recognizing an epitope within the 2F5 and 4E10 BNtAbs epitopes on gp41 (AB#31) with affinities in the nanomolar range was isolated. Importantly, it was shown to compete with 41 out of 42 gp160-reactive plasma samples from North-American and African HIV-1 positive patients, indicating that this antibody recognizes an epitope conserved in a large panel of isolates and might be suitable for diagnostic applications.

Since all vaccine candidates elicited antibodies reacting positively in HIV tests, Khurana *et al.* applied the phage display technology to identify HIV-1 epitopes susceptible to help discriminate between successfully immunized vaccinees and seroconverters [177]. They constructed a phage library displaying the full HIV-1 genome and screened it against the sera of newly seroconverted HIV-1 positive individuals. They identified conserved epitopes present in gp41 and in Gag p6 that were not part of the vaccine used at that moment and established a new detection test, named SELECTEST, that demonstrated over 99% selectivity and sensitivity for the early detection of seroconversion. HIV SELECTEST was able to detect antibodies against these epitopes in newly infected patients as early as 2 to 4 weeks after infection.

### 3.2. Inhibitors of Host Proteins

In parallel to the targeting of viral proteins, many efforts were undertaken to identify peptides, antibody fragments or modified ligands binding to the HIV-1 host proteins and impairing their interactions with the viral proteins (Table 5).

#### 3.2.1. Host receptors inhibitors

The HIV-1 host receptors CD4, CCR5 and CXCR4 are involved in the early steps of HIV-1 infection and thus represent valuable targets for the identification of antiviral peptides or neutralizing Abs. Moreover, these receptors display very low variability compared to the viral Env proteins facilitating the identification of neutralizing antibodies. Although the CD4 receptor plays a crucial role in the entry process, the only phage display biopanning assays reported to date targeted the chemokine receptors CCR5 and CXCR4. However, to circumvent the difficulties of purifying and immobilizing such complex receptors on a solid support without losing their native structure, biopanning procedures had to be adapted. In this regard, screening strategies using biopanning on living cells [178,179], proteoliposomes [180] or peptides derived from the receptors extracellular parts [181,182] were particularly successful.

##### 3.2.1.1. CCR5 Coreceptor

The CC chemokine receptor 5 (CCR5) is one of the two major HIV-1 coreceptors and binds three different endogenous chemokines CCL5 (RANTES), CCL4 (MIP-1β) and CCL3 (MIP-1α) which were reported to prevent R5-tropic HIV-1 entry. Interestingly, inhibition of CCR5 binding to HIV-1 provides an almost complete protection against R5-tropic viruses with only minor effects on the normal physiological functions of the cells [183].

The first biopanning experiment targeting CCR5 was performed using receptor embedded in paramagnetic proteoliposomes [180]. To create such proteoliposomes, magnetic beads were added to a mixture of synthetic lipids, a detergent-solubilized C9-tagged CCR5 receptor and a capture antibody, reconstituting membrane bilayers containing pure, native and properly oriented CCR5 receptor. These proteoliposomes were used in biopanning experiments with a human ScFv antibody library and several antibody fragments specifically binding to CCR5-expressing cells were identified. The same year, Steinberger *et al.* used the phage display technology to select and to humanize rabbit anti-CCR5 antibodies preventing the export of CCR5 to the cell surface [184]. Following rabbit immunization with a GST-Nterm CCR5 fusion protein, the authors constructed a phage displayed Fab library that was screened against the antigen initially used for the immunization. A phagotope (ST6) binding strongly and specifically to the immobilized antigen as well as to CCR5-positive cells was identified, expressed as a ScFv and humanized by successive replacements of the rabbit light and heavy chains by their human counterparts. One humanized antibody fragment, ST6/34, that retained the strong CCR5-binding capacity of the parental ST6 antibody was isolated from the screening of the intermediate libraries. When expressed as intrabody the ST6/34 scFV efficiently blocked the CCR5 expression at the cell surface.

In 2003, Hartley *et al.* developed another innovative and efficient strategy to identify CCR5 HIV-1 entry inhibitors based on CCR5 ligand variants selected by phage display [185] (reviewed in Chevigné *et al.* [191]. In this strategy, a phage library displaying randomly mutated and *N*-terminally extended CCL5 chemokine variants (xS#xSSx###-CCL5, where # represents either A, P, S or T) was constructed and screened on CCR5-expressing cells. Only intracellular phagotopes that had induced CCR5 receptor internalization were recovered. Two CCL5 variants (P1 = LSPVSSQSSA-CCL5 and P2 = FSPLSSQSSA-CCL5) were identified. These variants displayed a higher selectivity for CCR5 and had more potent HIV-1 inhibitory abilities than the wild-type CCL5 chemokine. Further characterization demonstrated that P2 acted as a CCR5 superagonist and potently induced intracellular CCR5 sequestration. P1 was less potent but significantly reduced CCR5-dependent intracellular calcium signaling. In a subsequent study, P1 and P2 variants were optimized by phage display biopanning to select for variants that retained the high anti HIV-1 potency of P1 and P2 but reduced CCR5-agonist activity [186]. Three successive generations of libraries (xxPx_3_Q#TP-CCL5, QGPPLMx_4_-CCL5 and QGPΨ$x_5_-CCL5 where Ψ represents G, L or P and $ represents G, L or M) were constructed and screened as previously described [185]. The three most interesting candidates (5P12-CCL5, 5P14-CCL5 and 6P4-CCL5) produced as soluble proteins displayed highly potent antiviral activities. Analogue 6P4-CCL5 acted as an agonist and sequestered CCR5, 5P12-CCL5 induced no signaling or receptor sequestration while 5P14-CCL5 induced CCR5 internalization without triggering G-protein signaling. Altogether these data demonstrated that antiviral activities of similar molecules identified through phage display screening can rely on various mechanisms of action.

Shortly after the identification of the P1 and P2 variants, Zhang *et al.* reported an alternative biopanning methodology relying on the use of small cyclic biotinylated peptides mimicking CCR5 extracellular loops (ECL1, ECL2 and ECL3) for the identification of CCR5-binding ScFvs [181]. A mouse phage-displayed ScFv library was incubated with biotinylated-ECL peptides and ECL-binding phages were specifically recovered using streptavidin-coated beads. Among the CCR5-binding ScFv identified, three clones (A1, B7 and L9) selected on cyclic ECL1 or ECL2 peptides inhibited R5- tropic HIV-1.

Screening of RPL was also applied to the discovery of small CCR5-blocking peptides through the targeting of receptor-expressing cells [178,179]. Vyroubalova *et al.* screened a partially randomized 10-mer phage library (CDx_3_KPCALLRYx_10_-PIII) using competitive elution with a CCL5 analogue (NNY-CCL5, 100 nM) and selected a unique peptide (ALLRYNPFYYLSFSP). This peptide was further optimized through *N*-terminal extension, exon shuffling and biopanning. By applying successive treatments consisting of a preselection using a low amount of NNY-CCL5 (100 pm) to discard low-affinity binding phages followed by classical alkaline elution using TEA and a competitive elution containing NNY-CCL5 (1 μM) they identified an extended peptide (LLDSTFFTADALLRYNPFYYLSFSP) inhibiting R5-tropic HIV-1 cell fusion with an IC_50_ of 5 μM. In parallel, Wang *et al.* screened a fully randomized 12-mer RPL using acidic elution and identified phagotopes binding specifically to CCR5-expressing cells and sharing the AFDWTFVPSLIL sequence [178]. In peptide and phage formats this sequence blocked the binding of the anti-CCR5 neutralizing 2D7 MAb and completely inhibited binding of the chemokine CCL5 to the receptor [179].

##### 3.2.1.2. CXCR4 Coreceptor

The CXC chemokine receptor 4 (CXCR4) is the second major HIV-1 coreceptor. CXCR4 binds only to one endogenous chemokine ligand (CXCL12) and is also expressed at the surface of numerous cancer cell types underlining its high value as therapeutic target [192,193]. Despite this importance and the relative success of phage biopanning on CCR5, only two recent studies reported the use of phage display to search CXCR4 inhibitors [182,187].

In 2010, Jahnichen *et al.* isolated llama-derived V_HH_ binding specifically to CXCR4 and inhibiting the entry of X4-tropic virus [187]. To select V_HH_ binding exclusively to functional and properly folded receptor, llamas were immunized with CXCR4-expressing HEK293T cells. A phage library was subsequently constructed from the PBMCs of immunized camelids and several phage clones inhibiting the binding of labeled CXCL12 chemokine to the receptor were identified. In particular, two V_HHS_ (238D2 and 238D4) showed low nanomolar affinity for the receptor and inhibited entry of X4 and X4/R5-viruses into different CXCR4_+_ cell types with IC_50_ values ranging from 10 to 100 nM. Dimerization of 238D2 and 238D4 to form biparatopic proteins increased their antiviral properties to IC_50_ values in the picomolar range. Epitope mapping revealed that the two V_HH_s inhibited CXCR4 mainly through binding to the second extracellular loop.

Very recently, we used a peptide corresponding to this particular extracellular loop (ECL2) as target to identify short CXCR4 antagonists [182]. By screening a non-immune phage library displaying the human HCDR3 peptide repertoire [194], several small peptides binding to the ECL2 peptide that specifically recognized CXCR4-expressing cells were identified. Notably, one of these HCDR3 peptides (TYPGRY) acted as a CXCR4 antagonist with potency in the micromolar range.

#### 3.2.2. Other Host Protein Inhibitors

In addition to targeting host receptors, a few studies reported the identification of peptides or antibody fragments directed against other host proteins such as cell surface determinants (CD) or intracellular enzymes [188,190,195,196]. The CD40 receptor, a member of the tumor necrosis factor receptor superfamily, and its CD40L ligand are involved in several biological processes including cell proliferation, activation and production of cytokines and chemokines. During HIV-1 infection, viruses were proposed to selectively downregulate or even deplete the pool of CD40L-expressing CD4+ T cells. In this context, antibodies binding to CD40 and restoring the HIV-1-induced CD40L downregulation might be of interest. In 2002, Ellmark *et al.* identified a set of anti-CD40 antibody fragments through biopanning of a human ScFv phage library against a biotinylated CD40 antigen [189]. When expressed as full-length IgG1 one antibody (clone B44) suppressed HIV-1 infection by a R5-tropic virus, most probably through induction of CC chemokine production [188]. More recently, the DDX3 protein, a cellular RNA helicase involved in RNA unwinding was shown to play important roles in HIV-1 replication. This protein presents a unique region (ALRAMKENG) responsible for high affinity binding to the HIV-1 RNA. Garbelli *et al.* carried out a biopanning experiment with a mix of linear 12-mer and linear and constrained 7-mer RPL against a peptide mimicking the DDX3 region interacting with HIV-1. They identified a 7-mer peptide (SDVPTQV) blocking the replication of an X4-tropic virus with an IC_50_ of 20 μM [190].

Besides therapeutic purposes, the phage display technology has also been applied for fundamental studies of host proteins. In particular, a study focusing on the roles and the diversity of the anti-CD34 autoimmune repertoire in the myelosuppression appearing in HIV-1 infected individuals was reported by Rubinstein *et al.* [195]. Using a substractive biopanning procedure from the immune repertoire of a HIV-1 seropositive patient, the authors selected Fab fragments binding specifically to the CD34 receptor. Sequencing and binding analyses of these antibody fragments demonstrated the heterogeneous origin of the anti-CD34 autoimmune repertoire and suggested that these autoantibodies might be generated through antigen-specific driven processes.

## 4. Phage Substrate

The use of phages for protease cleavage specificity profiling was first described by Matthews and Wells in 1993 [197]. This “phage substrate” approach relies on the use of phage particles to screen for enzyme substrates instead of classical binder selection. Protease cleavage profiling using phage takes advantage of the natural resistance of phage particles to proteolysis. Phage particles displaying random peptides are immobilized on a solid support and submitted to proteolytic elution to specifically liberate phages presenting peptides corresponding to the protease cleavage site. The phage substrate approach allows thus to rapidly determine the cleavage profile of a given protease and provides optimized substrate candidates which can be further used as leads for the development of specific inhibitors. Over the last two decades, phage substrate has been applied to a large variety of proteases including the HIV-1 protease (PR) [198,199]. The HIV-1 PR is a homodimeric aspartic protease responsible for nine critical cleavage steps within both the structural (Gag) and the non-structural (Gag/pol) polyproteins. The HIV-1 protease recognizes substrate residues encompassing P4 to P3′ positions (Schechter and Berger’s nomenclature), with the primary determinants from positions P2 to P2′ positions [200,201]. Interestingly, alignment of the nine natural substrate cleavage sites of the HIV-1 PR shows a high sequence diversity suggesting a broad proteolytic specificity. In 2000, Beck *et al.* reported the use of hexapeptide phage library to unravel the HIV-1 PR specificity and develop new protease inhibitors (Table 6) [199]. This library was constructed by fusing the Mab 3-E7 epitope upstream of the randomized sequences. Phages were first incubated with the HIV-1 PR and uncleaved phages were removed by addition of pansorbin cells. Biopanning selected for highly diverse sequences consistent with the suggested broad substrate specificity of the HIV-1 protease. However, none of the selected peptides corresponded to the HIV-1 polyproteins cleavage sites.

Nonetheless, several phage-displayed peptides were very efficiently cleaved by the HIV-1 protease. In peptide format, most of them displayed a *K*_m_ value lower than the one determined for a peptide mimicking the natural substrate at the matrix/capsid junction (IRKIL↓FLDG) (Table 6, italic). The most potent selected substrate (GSGIF↓LETSL) was cleaved 60 times more efficiently and had a *K*_m_ value of 5 μM *i.e.*, 260 times lower than the natural substrate (*K*_m_ = 1300 μM). Interestingly, the GS**G**IF↓LET**S**L substrate displayed only two residues (in bold) of the optimal cleavage site model designed based on the most frequently selected residues for each position (SGVY↓FVTS) (Table 7).

Sequences corresponding to the four most potent substrates were synthesized as peptidic transition state analogues and presented inhibitory activity in the nanomolar range (Table 6, underlined sequences). In 2001, the same authors applied the phage substrate approach to compare the relative specificities of the human (HIV-1) and feline (FIV) Immunodeficiency virus PRs [198]. Hexapeptides specifically processed by each of the two proteases as well as peptides cleaved by both enzymes were identified. Further mutational analysis of synthetic peptides derived from a sequence processed with the same efficiency by the two proteases (KSGVF↓VVNG) was performed to assess the influence of amino acid substitutions on the catalytic process of each PR (Table 6, underlined sequence). They showed that substitutions for a Val at position P2 or P2′ increased the cleavage by the HIV-1 protease whereas the introduction of a Val at position P1 was more favorable to FIV protease activity. In particular, the GSGVFΨ(CH_2_NH)VVNGL inhibitor identified in the first study was active against both PRs with different potencies and replacement of its amide group by a hydroxyethylene group resulted in a peptide with equivalent inhibitory activity towards both the HIV-1 and the FIV proteases.

## 5. Phage particles as HIV-1 Antigen Carriers

In parallel to epitope mapping, inhibitor discovery and enzyme profiling applications, bacteriophage particles were exploited as carriers for the development of anti-HIV-1 vaccines. For this particular purpose, phages are not used as affinity selection tools but rather to display antigens to the immune system, aiming to elicit specific neutralizing antibodies or/and efficient cytotoxic T-cell responses. Indeed, phages can also be considered as biologically inert particles characterized by a dense and repetitive organization capable of displaying a wide range of exogenous proteins at a precise valency and in a controlled manner. Moreover, phages were shown to be naturally immuno-stimulatory [202,203] and are particularly affordable, easy and rapid to produce and to administer in many animal models [204]. Altogether these characteristics make bacteriophages a suitable and valuable carrier for HIV-1 vaccine development.

Anti-HIV-1 vaccination trials to elicit humoral and cellular responses against various HIV-1 proteins such as the envelope proteins gp120 and gp41 as well as the RT and the p17 proteins [205–209] were conducted with a large diversity of bacteriophages including M13, T4, MS2 and lambda phages [205,210–213] (Table 8).

Numerous phage-vaccination trials using mimotopes identified on anti-HIV-1 antibodies were reported (See Tables 1–3). All these attempts aimed mainly at eliciting antibodies directed against the genuine epitope of an existing antibody. Besides these descriptions, a few studies were exclusively dedicated to the use of phage particles as vehicles to display fragments or complete HIV-1 proteins to the immune system to prime new immune responses.

The pioneer attempt was reported by Minenkova *et al.* in 1993 [205]. Immunization of rabbits with filamentous M13 phage particles displaying a small peptide (GEDRW) derived from the p17 Gag protein in fusion with pIII minor coat protein elicited the production of specific IgGs reacting with the natural p17 antigen as well as with the Gag precursor protein. Shortly afterwards, two studies reported that mice immunization with fd phages displaying the gp120 V3 loop fused to the pIII protein induced high titers of antibodies cross-reacting with V3 loops of different strains and featuring neutralizing activity [207,210]. The major coat protein pVIII of the fd phage (2700 copies per particles) was also investigated as scaffold protein for vaccination. Immunization with phages displaying a pVIII-fused peptide corresponding to residues 309–317 of the HIV-1 RT resulted in the induction of a specific cytotoxic T- cell response (CTL) against the displayed peptide. Priming nevertheless required a co-immunization with a T-helper epitope from the same protein (KDSWTVNDIQKLVGK) provided by either the same or a separate phage particle [206]. More recently attempts to prime CTL response were conducted with M13 phages displaying a mixture of thousands of variants of CTL epitopes (RGPGxAx_4_ or xGxGxAxVxI) derived from the gp120 V3 loop (residues 311–320; RGPGRAFVTI) presented in an immunoglobulin V_H_ domain scaffold [215]. Mice immunization provided potent and broad epitope-specific long lasting response (12 months) and effector memory T cells were induced. Moreover, recent studies demonstrated that mice immunized with these variable epitope libraries are capable of neutralizing half of the subtype B viral isolates used for challenge, including HIV-1 isolates which are known to be resistant to neutralization by several potent monoclonal antibodies [218].

Although they are versatile and easy to manipulate, filamentous phages present limitations for vaccination, and namely low display level and the limited size of the foreign peptides that can be incorporated without interfering with the natural functions of the phage coat proteins. Therefore, alternative bacteriophage models were explored. T4 phage and its two accessory capsid-decorating proteins SOC and HOC were demonstrated to be particularly efficient for high copy display of various large HIV-1 antigens [211,214,216]. In contrast to pIII and pVIII proteins, SOC and HOC present the major advantage of being dispensable for phage assembly yet of being able to strongly bind to the outer surface of the capsid, if added subsequently [219]. SOC and HOC self-associate to the capsid at a display level of 960 and 150 copies per capsid, a far higher density than that of filamentous minor coat protein (3 to 5 copies). HOC protein provides high-density display of single as well as multiple antigens and immunization of mice with the p24-HOC-T4 elicits strong humoral and cellular responses.

Moreover, fusion of different antigens to SOC and HOC offer the possibility to develop multicomponent HIV-1 vaccine particles [216] T4 phage displaying V3 loop of the gp120 protein fused to SOC protein was reported to elicit antibodies capable of recognizing the native antigen [214]. Further study demonstrated that HOC protein can also be used to display various HIV-1 antigens including the p24, Nef or a trimeric peptide derived from the *C*-terminus of gp41 [216]. More recently, lambda phage and its decorating capsid protein were used for dense display of glycosylated mammalian cell-derived trimers of gp140 protein [213]. Lambda phage provided a display level of 30 copies of gp140 trimer per particle, a 20-fold higher display than observed on native HIV-1 virions (14 ± 7 spikes per virion) [220]. Rabbit immunization trials were rather disappointing and higher antibody titers were elicited in animals receiving soluble oligomeric gp140. These results were proposed to be due to the sequential immunization process and to the strong and immunodominant response against phage capsid proteins, which is most probably boosted upon sequential immunization, and could lead to a decrease of the humoral immune response to the displayed antigen.

Virus like particles (VLP) derived from the MS2 bacteriophage coat protein displaying the V3 loop of gp120 and devoid of phage genome were also reported [212]. Although characterized by a low display level (90 copies per VLP) these particles were nevertheless able to elicit high titers of specific antibodies when injected in mice and provided neutralizing activity at 1/10 sera dilution. More recently, PP7 phage-derived VLPs displaying the V3 loop of gp120 were used to immunize mice providing high-titer antibody response [217].

## 6. Conclusions and Future Challenges

AIDS was first described in 1981 [1], and two years later HIV-1, its causative agent, was isolated [2]. The phage display technology was first published almost concomitantly in 1985 [12]. Both protagonists lived parallel lives until 1991, when Burton *et al.* identified a human Fab recognizing the CD4-binding site of HIV-1 gp120 through phage display [41]. This first antibody, b12, turned out to be one of the rare BNtAbs characterized to date, and many studies are still ongoing to set up scaffolds presenting its epitope in a conformation capable of eliciting Abs with b12-like HIV-1 inhibiting properties. This article was the starting point of a dense literature exploiting the different applications of the phage display technology to gain as much knowledge as possible on the HIV-1 infection process.

The complex hide-and-seek game between HIV and the host immune system and the absence of an efficient vaccine candidate emphasizes the need to better comprehend the HIV-1-related immune response and to identify antiviral molecules. HIV-1 is to the best of our knowledge the only pathogen to which all four applications of the phage display technology described in this review—*i.e.*, epitope mapping, inhibitor discovery, substrate presentation and carrier phage- have been applied. Parallel improvements in the technology on one hand, and crucial discoveries in the HIV-1 field on the other hand, most likely account for such a tight interplay. The phage display technology applied to HIV-1 has provided precious knowledge concerning the regions recognized by the Ab repertoire of immune patients, and allowed to identify immunodominant regions. It has also allowed to map the epitopes of numerous monoclonal antibodies directed against viral and host proteins, and, most importantly, discontinuous epitopes. Alternative phage display biopanning substractive procedures such as epitope masking or competitive antigen panning also precisely highlighted non-immunodominant regions of the HIV-1 antigen, which had remained occult using classical biopanning approaches. Although the antibodies identified through epitope-masking are mostly devoid of neutralizing activity, this strategy led to the identification of Fabs binding with high affinity to specific targets. Taking advantage of this affinity, approaches aiming at engineering bispecific antibodies, coupling these inhibitors to toxins, or multimerizing them to increase their binding avidity could lead to the development of antiviral compounds or more efficient vaccine candidates.

Although highly informative, immunization trials performed with mimotopes/phagotopes selected through phage display remained altogether rather disappointing as no long-lasting neutralizing response was reported, and provided yet further proof that antigenicity does not necessarily imply immunogenicity [221]. Among the bottlenecks in the field of HIV-1 vaccine research and development are the weak immunogenic properties of the identified mimotopes or native antigens. Whether the difficulty of eliciting and/or isolating strong or broad neutralizing antibodies from HIV-1-infected patients, be they normal Progressors or LTNPs, is a caveat of phage display or a real reflect of the restricted humoral response in fighting HIV, or to both, remains to be established. Nevertheless, these discrepancies are not exclusive of HIV-1 mimotopes and were observed with other antigens.

From a fundamental prospect, the use of phage-displayed IgM libraries to model Abs close to the germline Abs allowed to elegantly expore the poorly characterized determinants of the initial antiviral immune response. Phage display also contributed to a better knowledge on the structure of the diverse HIV-1 proteins as well as a gain of insight on non-structural proteins involved in replication mechanisms. A highlight example would be the phage substrate approach which allowed the precise characterization of the HIV-1 protease cleavage specificity and thereby provided valuable inhibitor candidates. Unraveling viral replication steps or protein interactions led to exploit phage display to engineer various types of inhibitors targeting either viral or host proteins.

In parallel, phage display allowed the identification of various types of inhibitors targeting either viral or host proteins. Nevertheless, only a few strong antiviral candidates with broad neutralizing activities and/or potencies in the nanomolar or even picomolar range were identified with the phage display technology while most of them presented inhibiting activities that ranged at the best in the micromolar range and could not be further exploited.

**Figure 5:**
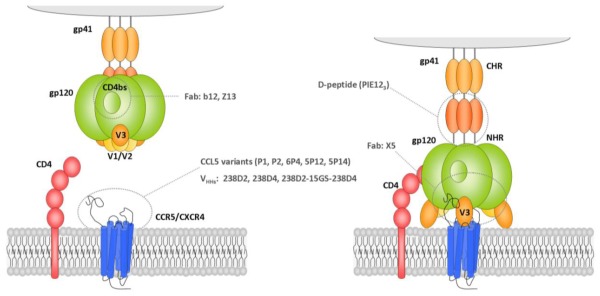
HIV-1 entry inhibitors identified by phage display and their target. Potent HIV-1 inhibitors blocking key steps in the entry process were identified using the phage display technology. These inhibitors target: the CD4 binding site (Fab b12 and Z13), the coreceptors CCR5 (CCL5 variants) or CXCR4 (V_HH_ 238D2 and 238D4), the CD4-induced epitope of gp120 (Fab X5) or the heptad repeat region of gp41 (peptide PIE12_3_).

Some of the most potent inhibitors originated from Fab libraries derived from asymptomatic HIV-1-infected patients whose reactivity against HIV-1 was previously assessed (b12, X5, Z13) or from immunized camelids (V_HH_ anti CXCR4). Other inhibitors were identified from semi-synthetic (ligand analogues of CCL5) or randomized peptide libraries (d-peptides: PIE12_3_) and their affinity was improved through secondary libraries. These inhibitors were selected against different targets (Env proteins, coreceptors) by biopanning carried out using different types of support (immobilized proteins, cells, peptides), illustrating the power and versatility of the phage display technology [41,83,85] (Figure 5).

Despite the variable success of the phage display technology *per se* in developing a direct antiviral agent or immune response, this technology continues to contribute precious and otherwise inaccessible developments in the field of HIV-1 research, mainly owing to the ability of phages to display a high density of either single antigens or libraries of antigens/mimotopes. To this regard, phages are being extensively explored to increase immune responsiveness. The latest developments include exploiting the phage as DNA vaccine carrier or hybrid phage and pursuing the symbiotic interplay between phage display and HIV-1.

## Figures and Tables

**Table 1:** Epitope mapping of monoclonal antibodies directed against viral epitopes.

Target	Library	Target presentation	Mimotopes	Epitopes	Additional	Vaccination attempts	Results	Reference
MAb447-52D	15-mer RPL 2 sublibraries (Cx_5_GPxRx_5_C, LLx_5_GPxRx_5_LL)	NA Ab captured on polystyrene beads	GPxR	gp120 V3 loop	NA	Rabbit immunization with 1 peptide	NAbs induced	[17,18]
MAb 58.2	20-mer RPL	Biotinylated Ab captured on streptavidin-coated plates Microwells	(Y/L)(V/L/I)GPGRxF	gp120 V3 loop	Characterization of non-essential aminoacids in the epitope using a peptide array	NA	NA	[19]
MAb 19B	15-mer RPL	Ab captured on polystyrene beads	xIx_4_GxxFYxT	gp120 V3 loop	*Type II β* turn structure of the epitope	NA	NA	[18,20]
MAb 1001	30-mer RPL	Ab coated onto microwells	(R/K/H)xGR	gp120 V3 loop	Peptides fused to *E. coli* AP. Affinities for the Ab determined by SPR	NA	NA	[21]
MAb 110-A MAb 19.26.4	6-mer RPL	NA	Hyx**R**GP x**Q**(**R**/K)GP	gp120 V3 loop	QR insertion characteristic of the LAI isolate	NA	NA	[22]
MAb 268	6-mer RPL	Biotinylated Ab captured on streptavidin-coated beads	268.1 (HLGPGR), 268.2 (KAIHRI)	gp120 V3 loop	NA	Rabbit immunization	gp120-specific Abs	[23]
MAb 2F5	15mer	Ab captured on polystyrene beads	ELDKW, D(K/R)W	ELDKWA (gp41 MPER)	2F5 affinity for gp41 and gp41 peptide determined by SPR	NA	NA	[24]
MAb 2F5	17 constrained and linear RPL, x_12_-AADKW and AADKW-x_12_ sublibraries	Biotinylated Ab captured on streptavidin-coated beads	3 peptides (AADKW-x_12_)	ELDKWA (gp41 MPER)	Ala substitutions and deletions studies	NA	NA	[25]
MAb 2F5	12-mer and 7-mer-c RPL	Ab coated onto microwells	DKWA, LDKWA	ELDKWA (gp41 MPER)	Epitope specificity depends on the structural context of the library	Mice and rabbit immunization	Inhibition of cell fusion	[26]
MAb GV1A8 MAb GV4D3	20-mer RPL	Rabbit anti-mouse IgG, Fc-specific Ab coated on Petri dishes	HxxIxxLW Nx_3_WxxD	C1 domain of gp120	Pepscan analysis, quantitative binding analysis	NA	NA	[27]
MAb ID6	12-mer, 7-mer, 7-mer-c RPL	Mab captured on rat anti-mouse Ab coated microwells	TxxFxxWxxD, FxDWxF	C1 domain of gp120	ADCC-inducing Ab	NA	NA	[28]
MAb b12	11 RPL 2 sublibraries (x_7_**SDL**x_3_**CI** and x**C**xx**SDL**x_3_**CI)**	Biotinylated IgG1b12 captured on avidin-coated microwells	REKRWIF**SDL**THT**CI**, T**C**LW**SDL**RAQ**CI** B2.1 (HERSYMF**SDL**ENR**CI)**	gp120 CD4 binding site	Competition with gp120 for the binding to IgG1b12, determination of the in-solution binding affinity.	(2) Phage B2.1 immunization of mice and rabbits	Anti-peptide Abs elicited	[29,30]
MAb b12	x_10_ALLRYx_10_, x_3_(M/V)WSDx_3_ and xLXVWxDExx RPL	Ab coated onto tubes	GLLVWSDEL	gp120 CD4 binding site	NA	Mice immunization	Env-specific Abs	[31]
MAb b12	12-mer-c RPL	Ab captured on Protein G-coated Petri dishes	WSDL	gp120 CD4 binding site	Prediction of epitope clusters with Mapitope	NA	NA	[32]
MAb 5145A	9-mer and 10-mer-c RPL	Ab coated onto Petri dishes	AECGPAEPRGAWVC, AECGPYEPRGDWTCC	gp120 CD4 binding site	NA	Rabbit immunization with peptides-HSP fusion constructs	gp120-specific Abs elicited, recognizing a different epitope	[33]
MAb 803-15.6	7-mer RPL	Ab coated onto microwells Ab coated onto microwells with gp120	AxxKxRH	gp120 residues 502–508	Quantitative binding analysis	NA	NA	[34]
MAb 17b MAb 13b5 MAb CG10	12-mer-c RPL	Ab captured on Protein G-coated Petri dishes	NA	(3) gp120 CD4-induced epitope, p24	Prediction of epitope clusters with Mapitope	NA	NA	[32]
MAb A32 MAb 50-69	15-mer RPL	Ab captured on polystyrene beads	W **W**GCx(K/R)xLxC, FGx**W**FxMP	CD4- induced epitope gp41 ID GKLIC	NA	NA	NA	[18]
MAb 2G12	Set of RPL	Ab captured on protein A-coated beads	2G12.1 (ACPPSHVLDMRSGTCL)	Glycosylated gp120	NA	Rabbit immunization	No HIV-specific Abs	[35]

NA: data not available, Hy: any non-aromatic AA, underlined: AA tolerating substitutions, AP: alkaline phosphatase.

**Table 2:** Epitope mapping of polyclonal antibodies directed against viral epitopes.

Library	Biopanning support	Samples	Target	Biopanning procedure	Mimotopes	Epitopes	Additional	Vaccination attempts	Results	Reference
9-mer, 9-merc RPLs	Tosylactivated beads coupled to Anti human IgG, Fc-specific Ab	LTNP (serum)	IgG	Panning on one LTNP, reactivities assessed on a second LTNP	3 linear, 7 putative conformational	Linear: gp41 ID GKLIC region, gp120 V1 and C2 regions	Reactivities of positive clones assessed on 22 LTNP, 25 HIV, 50 HD. Affinity purification of Nabs with phages	Phage immunization of C57BL and BalB/c mice	NAbs induced	[50]
12-mer-c RPL	Protein G coated on Petri dishes	HIV + (serum)	IgG	Panning on HIV+ serum IgG	4 linear, 2 unassigned	Linear: gp41 ID GKLIC region	Reactivities of the clones assessed on the sera of 30 HIV+	NA	NA	[52]
12-mer, 7- mer, 7-mer-c RPLs	Microwells coated with purified IgG	HIV+ (HAART)	IgG	Panning on purified HIV+ IgG, before and after HAART	Linear, CxxKxxC	Linear: gp41 ID GKLIC region	Reactivities of 1 insert with IgG of 22 HIV+	Phage immunization of C57BL/6J mice	Abs against epitope	[53]
12-mer, 7- mer, 7-mer-c RPLs	Tosylactivated beads coupled to Anti human IgG, Fc-specific Ab	LTNP (plasma)	IgG	Panning on pool of 8 LTNP plasma, negative selection on HIV - plasma pool	160 linear, 124 putative conformational, 160 unassigned	gp120 V3 loop, gp41 GKLIC, gp120 MPER	Reactivities with plasma of 7 HIV+ plasma	Phage immunization of mice	NAbs induced	[54]
12-mer, 7- mer, 7-mer-c RPLs	Tosylactivated beads coupled to Anti monkey IgG Ab	Clade C SHIV-infected rhesus macaque	IgG	Panning on one SHIV + plasma, negative selection on SHIV - monkey plasma pool	72 linear, 6 putative conformational	gp120 V2, V3, C-term, gp41 GKLIC, MPER	Reactivities of inserts tested as fusion proteins	DNA prime-phage boost of BALB/c mice	NAbs induced	[55]
12-mer RPL	Tosylactivated beads coupled to Anti human IgG, Fc-specific Ab	HIV subtype CRF02AG-infected plasma with 4E10-like BNtAbs	IgG	Panning on pooled longitudinal samples of one HIV + plasma, negative selection on HIV - plasma pool	Linear (SLxxLRL, KxWWxA, Kx_3_IGPHxxY)	gp41 MPER and LLP2 regions; gp120 C1 and V3 regions	NA	NA	NA	[56]
12-mer, 7- mer, 7-mer-c RPLs	Tosylactivated beads coupled to Anti human IgG, Fc-specific Ab	HIV subtype A-infected plasma with 4E10-like BNtAbs	IgG	Panning on pooled longitudinal samples of one HIV + plasma, negative selection on HIV - plasma pool	38 linear, 22 putative conformational (Kx_3_Hx_3_Y, KxxHxGPx_3_F, CxGxLxCTxNxP)	gp41 ID epitope; gp120 V2 and V3 regions	Competition with rgp120/gp140 for antibodies binding; peptide neutralization inhibition assays	NA	NA	[57]
DNAse-fragmented Gag DNA	Purified IgG captured on microwells	Serum of rabbits immunized with p24	IgG	Panning on rabbit anti p24 IgG	p24 fragments	N- and *C*-terminus of p24	Reactivities of inserts tested as proteins in fusion with GST	NA	NA	[58]

NA: data not available.

**Table 3:** Epitope mapping of monoclonal antibodies directed against host proteins.

Target	Library	Target presentation	Mimotopes	Epitopes	Additional	Vaccination attempts	Results	Reference
MAbs 3A9, 5C7	7-mer, 7-mer-c, 9-mer-c, 12-mer RPL	Phage-antibody complexes captured on beads coated with anti-mouse IgG Ab	CHA**SIYDFG**SC, C**PHW**LR**DLR**VC	CCR5 *N-*terminus (SIYD) and ECL1 (FG) CCR5 *N*-terminus (P), ECL1 (HW) and ECL3 (DLR)	Reactivities assessed against inserts synthesized as peptides, binding and competition assays with peptides. Entry inhibitor	NA	NA	[62,63]
MAb 2D7	15-mer RPL	Ab coated onto Petri dishes	M23 (FCALDGDFGWLAPAC)	CCR5 ECL2	Neutralization of HIV-1 infection	NA	NA	[64]
MAb 2D7	12-mer RPL	Ab coated onto microwells	EW**QKEGL**V**TL**WL	CCR5 ECL2	NA	Rabbit immunization	NAbs with 2D7-like functions	[65]
MAb MHM23	9-mer, 9-mer-c RPL	Phages-Ab complexes captured on streptavidin-coated dishes	PPFxYRK	CD18 (AA 200–206)	Inhibition of HIV-1-induced syncytium formation	NA	NA	[66]

NA: data not available.

**Table 4:** HIV-1 protein inhibitors selected by phage display.

Target	Library	Target presentation	Phagotopes	Affinity (K_D_)	Inhibition (IC_50_)	Virus isolates/clades	Additional	Reference
gp120 (CD4 BS)	LTNP Fab	Recombinant gp120	b12	<10 nM	~20 nM	MN, IIIb	NA	[41,42,70]
gp120 (CD4 BS)	Fab (CDR walking of b12 Fab)	Recombinant gp120	3D3	15 pM	~0.1–0.9 nM	MN, IIIb	3B3 ScFv engineered as fusion immunotoxin	[71–73]
gp120 (CD4 BS)	LTNP Fab	Recombinant gp120	L78	4–300 nM	~2 ug/mL	MN, IIIb	epitope-masking strategy	[74]
gp120 (CD4 BS)	ScFv (lupus patients) Light chains (lupus patients)	Recombinant gp120	JL413 SKL6	NA	0.1–25.6 μg/mL NA	23135, SF-162, ZA009, BR004, Ug246 NA	gp120-hydrolyzing Abs	[75,76]
gp120 (CD4 BS)	V_HH_ (immunized llamas)	Recombinant gp120	A12 D7 C8	0.1–1 nM	0.003–38 μg/mL	A, B, C, D, CFR02_AG and CRF07_BC	sublibrary engineered to increase potency	[77,78]
gp120 (CD4 BS)	CD4 V1 and V1-V2 variants	Recombinant gp120	E6, B6, 22, F8, D11	NA	0.2–1 μg/mL	BH10	NA	[79]
gp120 (V3 loop)	MAb 447-52D-derived ScFv	V3 loop peptide	402P5H7	0.28–3.1 nM	NA	MN	V_L_ shuffling and HCDR3 “spiking” of MAb 447-52D	[80]
gp120 (V3 loop)	LTNP Fab	Recombinant gp120	DO142-10 Fab loop 2	11 nM 1.9 nM	0.2–8 μg/mL 1–5 μg/mL	MN, IIIb	epitope-masking strategy	[70,81]
gp120 (CD4-i epitope)	7-mer, 9-mer-c and 12-mer RPL	Recombinant gp120	12p1	NA	1.1–1.6 μM	YU2	NA	[82]
gp120 (CD4-i epitope)	HIV-1 infected FDA-2 patient FAb	gp120-CD4-CCR5 complexes	X5	nanomolar	0.29–125 μg/mL	A, B, C, D, F and G	NA	[83]
gp120 (CD4-i epitope)	7-mer, 7-mer-c and 12-mer RPL	sCD4-89.6 Env on retroviral particles	XD3	NA	50 μM	NL4-3, D117III	NA	[84]
gp41 MPER	HIV-1 infected FDA-2 patient FAb Z13-derived Fab	MPER peptide	Z13 Z13e1	NA	>40–190 μg/mL <2–190 nM	B, C and E	NA	[85] [86]
gp41 Heptad Repeats	xxCDYPEWQWLCxx naïve human ScFv Fab “Tyr/Ser” 7-mer and 12-mer RPL	IZN17 5-Helix, IZN36 5-Helix N34(L6)C28	PIE12-trimer D5 13 clones JCH-4	NA	0.5 nM 93–1750 nM – 9 μg/mL	A, B, C, D, F, G, CRF at least 5 isolates – HXB2	(1) D-peptide	[87–89] [90,91] [92] [93,94]
gp41 Heptad Repeats	(1), (2) Naïve human Fabs (3) Fab 3674-derived Fabs	N35CCG-N13, NCCG-gp41 NCCG-gp41, 6- HB NCCG-gp41	8 Fabs Fab 3674 Fabs 8060, 8066 and 8068	– 97 nM 7–25 nM	6–60 μg/mL 450–2100 nM 26–1400 nM	LAV at least 5 isolates 7 isolates	NA	[95] [96–98]
gp41 Heptad Repeats	Rabbit ScFv, HIV-1-infected FDA-2 patient FAb HIV-1-infected patient Ab library	N35CCG-N13 gp140, gp120 as competitor	8K8, DN9 M44, M46, M48	NA	50–500 nM NA, 1.5–25 μg/mL, 9–22 μg/mL	13 isolates 4 isolates	(2) competitive antigen panning	[99] [100–102]
Vpr	7-mer RPL human ScFv	GST-Vpr Vpr *N*-terminus (17–34)	WxxF consensus ISSD, AFMKSGKRFIH, HFHYKGKLQTF	NA	NA	NA	cotransfection of WxxF-CAT constructs and Vpr inhibition of Vpr-mediated nuclear import	[103] [104]
Int	7-mer RPL	Recombinant Int	FHNHGKQ	NA	NA	NA	Inhibition of strand transfer activity	[105]
Tat Cyclin T1 TAR RNA	HIV-1-infected patient Fab naïve human ScFv 12-mer RPL	Tat AA 22–33 cyclin box domain, TRM TAR RNA	3 Fabs 3R6-1 57-mers	10–1000 nM 107 nM 420–550 nM	NA	NA	(2) inhibition of Tat-mediated transactivation and HIV-1 replication when transfected	[106] [107] [108]
NCp7 Psi RNA	9-mer-c RPL RPLs	NCp7 AA 30–52 psi RNA	PPx(D/E)R motif HWWPWW motif, SYQWWWHSPQTL motif	NA	NA	NA	(2) reduction of virus release by infected cells	[109] [110–112]
Nef Vif	mouse ScFv, immunized llamas V_HH_, human ScFv 12-mer RPL	recombinant Nef, Nef CTL 138-10/A24 recombinant Vif	SdAb19, ScFV3, ScFv27 PxP motif	(1) 2nM	NA	NA	(2) inhibition of HIV- 1 replication	[113–115] [116,117]
RT	mouse Fab human ScFv	Recombinant RT	5F, 5G F-6, 6E9, 5B11	NA	NA	NA	Inhibition of RDDP and/or DDDP activity	[118,119] [120]
Rev	HIV-1-infected patient Fab 15-mer RPL immunized llamas V_HH_ chimeric rabbit/human Fab	Rev	2 Fabs p1, p3, p19 Nb190 SJS-R1	NA	NA	NA	NA	[106,121–124]
Gag	12-mer RPL	CA protein (AA 162–190)	CAI	NA	NA	NA	*In vitro* inhibition of capsid assembly	[125]

NA: data not available.

**Table 5:** Host protein inhibitors selected by phage display.

Target	Library	Target presentation	Biopanning procedure	Phagotope	Affinity	Inhibition	Virus isolates/clades	Additional	Reference
CCR5	Human ScFv	Liposome	Five rounds	NA	NA	NA	NA	NA	[180]
CCR5	Human V_H_-Rabbit ST6 HCDRs	CCR5 Nterm-GST fusion	Four rounds, acidic elution	Rabbit ST6 Human ST6/34	2.7 nM 8.5 nM	NA	NA	Inhibit CCR5 export when expressed as intrabodies	[184]
CCR5	Randomized and extended CCL5 chemokine	HEK-CCR5, CHO-CCR5	Recovery of internalized phages, acidic elution	CCL5 P1-CCL5 P2-CCL5	IC_50_ = 4.1 nM IC_50_ = 2 nM IC_50_ = 0.2 nM	NA IC_50_ = 7.0 nM IC_50_ = 0.6 nM	BaL	P1: triggers reduced signaling P2: superagonist inducing CCR5 sequestration	[185]
CCR5	Randomized and extended CCL5 chemokine	HEK-CCR5, CHO-CCR5 cells	Recovery of internalized phages, acidic elution	6P4-CCL5 5P12-CCL5 5P14-CCL5	NA NA NA	IC_50_ = 21 pM IC_50_ = 28 pM IC_50_ = 26 pM	BaL	Superagonist + sequestration No signaling + no sequestration Internalization + no signaling	[186]
CCR5	Mice and rats ScFV	ECL1, ECL2, ECL3 biotinylated peptides	Alkaline elution	A1 (ECL1) B7 (ECL2) L9 (ECL2)	NA NA NA	25% ^a^ 35% ^a^ 25% ^a^	BaL	All active ScFv were selected on cyclic peptides	[181]
CCR5	12-mer RPL	CHO-CCR5 cells	Four rounds, acidic elution	AFDWTFVPSLIL	IC_50_ = 2.5 μM	NA	NA	Antagonist of CCR5	[179]
CCR5	Partially randomized CDx_3_KPCALLRYx_10_ x_10_ALLRYNPFYYLSF SP	CHO-CCR5 cells	Four rounds, competitive elution Four rounds, preselection, alkaline and competitive elutions	ALLRYNPFYYLSFSP LLDSTFFTADALL RYNPFYYLSFSP	NA	No inh IC_50_ = 2.5 μM	ADA	Does not act as CCR5 agonist or antagonist	[178]
CXCR4	Llamas immunized V_HH_	HEK-CXCR4 cells	Two rounds and counterselection	238D2 238D4 238D2-15GS-238D4	K_I_ = 10 nM K_I_ = 6 nM K_I_ = 0.3 nM	IC_50_ = 30 nM IC_50_ = 40 nM IC_50_ = 0.2 nM	NL4.3	Inhibit chemotaxis and mobilize stem cells	[187]
CXCR4	Non immunized HCDR3	Linear ECL2 peptide	Four rounds, acidic elution	YYCARDRGGTYPGR YWCQG	K_D_ = 5 μM	NA	NA	Antagonist of CXCR4	[182]
CD40	Human ScFv	Biotinylated CD40-Fc fusion protein	Four rounds, trypsin elution	B44	K_D_ = 60 nM	3–4 fold reduction of infection	R5- tropic	Activates normal B cells, Does not block CD40- CD40L binding	[188,189]
DDX3	Mix of 12-mer and 7- mer RPL	His tagged ALRAMKENGR peptide	Acidic elution	INS1: SDVPTQVGGRRRRRRRRR	NA	IC_50_ = 20 μM	LAI	Homologies to XPO1/CRM1	[190]

NA: data not available. A: tested at a unique concentration of 100 μg/mL.

**Table 6:** Phage substrate analysis of the HIV-1 protease cleavage specificity.

Target	Library	Biopanning	Natural substrates P4-P3-P2-P1--P1′-P2′-P3′-P4′	Selected substrates	*K*_cat_/*K*_m_ (μM^−1^s^−1^)	Derived Inhibitors	*K*_I_ (nM)	Reference
HIV-1 protease Q7K	Random hexapeptides fused to the MAb 3-E7 epitope and displayed on Fd phage	5 rounds with progressive decrease of the protease concentration and contact time	*(RT/IN) RKIL↓FLDG* (MA/CA) SQNY↓PIVQN (CA/P2) ARVL↓AEAM (P2/NC) ATIM↓MQRG (P1/P6) PQNF↓LQSR (in P6) KELY↓PLTS (TF/PR) SFNF↓PQIT (PR/RT) TLNF↓YVDG (in RT) AETF↓YVDG	GSGIF↓LETSL	1.3			[199]
				GSGVF↓VEMPL	3.9	GSGIFΨ (CH_2_NH)LETSL	5	
				GSGVF↓VVNGL	18	GSGVFΨ (CH_2_NH)VEMPL	18	
				GSGLF↓TEYGL	0.002	GSGVFΨ (CH_2_NH)VVNGL	100	
				*IRKIL↓FLDG*	0.002	GSGLFΨ (CH_2_NH)TEYGL	200	
HIV-1/FIV proteases		4 rounds with progressive decrease of the protease concentration			**Protease**		**IC_50_(nM)**	[198]
				SGNF↓VVNGLVK	HIV-1 only			
				KSGVF↓VENGLVK	HIV-1 only			
				KSGVF↓VQNGLVK	HIV-1 only			
				KSGNFVVN↓GLVK	FIV only			
				KSGV↓SVNGK	FIV only			
				KSGVFHVN↓GLVK	FIV only	GSGVF Ψ (CH_2_OH)VVNGL	HIV/FIV	
				KSGVFQVN↓GLVK	FIV only	GSGVF Ψ (CH_2_NH)VVNGL	160/5000	
				KSGVF↓VVNGLVK	HIV-1/FIV		700/400	
				KSFVF↓VVNGLVK	HIV-1/FIV			
				KSGIF↓VVNGLVK	HIV-1/FIV			
				KSGVF↓HVNGK	HIV-1/FIV			
				KSGVF↓QVNGLVK	HIV-1/FIV			
				KSGVF↓VVQGLVK	HIV-1/FIV			
				KSGVF↓VVTGLVK	HIV-1/FIV			

Arrow denotes cleavage sites in the natural or the selected substrates sequences. Underlined substrates correspond to sequences used to derived inhibitors. Italic sequences correspond to natural substrate (matrix/capsid junction) used as reference for the assessment of the cleavage efficiently. Ψ: amid-reduced bound.

**Table 7:** HIV-1 protease specificity model.

P4	P3	P2	P1	P1′	P2′	P3′	P4′
S_11_	G_11_	V_5_	Y_5_	F_4_	V_5_	T_4_	S_3_
		I_3_	F_4_	V_3_	E_4_	S_3_	T_2_
		N_3_	M_2_	L_3_	A_1_	M_1_	G_2_
				H_1_	Q_1_	E_1_	P_1_
						N_1_	N_1_
						Q_1_	D_1_
							A_1_

The model was build based on the 11 most efficiently cleaved phage substrates. Underscript numbers represent the frequency of appearance of the amino acid at a given position.

**Table 8:** Use of phage particle as HIV-1 antigen carrier.

Phage	Type	Carrier protein	HIV-1 antigen	Display level	Animal Model	Reference
M13	Filamentous	pIII	p17 (GEDRW)	3 to 5 copies/phage	Rabbit	[205]
fd	Filamentous	pIII	V3 loop of gp120	3 to 5 copies/phage	Mice	[207]
fd	Filamentous	pIII	V3 loop of gp120	3 to 5 copies/phage	Mice	[210]
fd	Filamentous	pVIII	RT (ILKEPVHGV)	2700 copies/phage	Human cells	[206,214]
M13	Filamentous	pVIII	V3 loop variants	2700 copies/phage	Mice	[215]
T4	Lytic/Icosahedral	SOC	V3 loop of gp120	960 copies/phage	Mice	[214]
T4	Lytic/Icosahedral	HOC	p24	150 copies/phage	Mice	[216]
MS2	VLP/Icosahedral	A-B loop	V3 loop of gp120	90 copies/VLP	Mice	[212]
PP7	VLP/Icosahedral	A-B loop	V3 loop of gp120	-	Mice	[217]
Lambda	Lytic/Icosahedral	gpD	gp140 trimer	30 copies/phage	Rabbit	[213]

## Abbreviations

AA: amino acids
Ab: Antibody
ADCC: Antibody-Dependent Cellular Cytotoxicity
AIDS: Acquired ImmunoDeficiency Syndrome
ARM: Arginine Rich Motif
BNtAb: Broadly Neutralizing Antibody
BS: Binding Site
BSA: Bovine Serum Albumin
CCR5: CC chemokine Receptor 5
CD: Cluster of Differentiation
CDR: Complementarity Determining Region
CH: Constant Heavy chain domain
CHR: *C*-terminal Heptad Repeats
CTL: Cytotoxic T-Lymphocytes
CXCR4: CXC chemokine Receptor 4
DDDP: DNA-Dependent DNA Polymerase
ECL: ExtraCellular Loop
Fab: Fragment antigen-binding
FIV: Feline ImmunoDeficiency Virus
FR: Frameworks
Gag: group-specific antigen
gp: glycoprotein
HAART: Highly Active Anti-Retroviral Therapy
HB: Helix Bundle
HcAb: Heavy-chain only Antibody
HCDR: Heavy-chain Complementarity Determining Region
HIV-1: Human ImmunoDeficiency Virus
HLA: Human Leukocyte Antigen
HOC: Highly antigenic Outer Capsid protein
IC_50_: Inhibitory Concentration 50
ID: Immuno Dominant
ID_50_: Inhibitory Dose 50
Ig: Immunoglobulin
IN: Integrase
KD: dissociation constant
KLH: Keyhole Limpet Hemocyanin
LTNP: Long-Term Non-Progressor
MAb: Monoclonal Antibody
MHC: Major Histocompatibility Complex
MPER: Membrane Proximal External Region
NC: NucleoCapsid
NCp: Nucleocapsid protein
Nef: Negative factor
NHR: *N*-terminal Heptad Repeats; NLS, Nuclear Localization Signal
NNY: n-nonanoyl
NtAb: Neutralizing Antibody
PAb: Polyclonal Antibody
PBL: Peripheral Blood Lymphocytes
PBMC: peripheral blood mononuclear cell
PHI: PreHairpin Intermediate
PIC: PreIntegration Complex
PIE: Pocket-specific Inhibitor of Entry
PR: Protease
psi: packaging signal
RDDP: RNA-Dependent DNA Polymerase
Rev: Regulator of virion expression
RPL: Randomized Peptide Library
RT: Reverse Transcriptase
ScFv: Single-chain variable fragment
SOC: Small Outer Capsid protein
SPR: Surface Plasmon Resonance
TAR: Transactivation Response element
TCR: T-Cells Receptor
TRM: Tat/TAR Recognition Motif
UDG: Uracyl DNA Glycosylase
V_H_: Variable Heavy chain domain
V_HH_: Variable Heavy-chain domains of Heavy-chain only Antibodies
Vif: Virion infectivity Factor
V_L_: Variable Light chain domain
vpr: Viral Protein of Regulation
VLP: Virus-Like Particle.
